# Meta-Analysis of Interrater Reliability of Supervisory Performance Ratings: Effects of Appraisal Purpose, Scale Type, and Range Restriction

**DOI:** 10.3389/fpsyg.2019.02281

**Published:** 2019-10-18

**Authors:** Jesús F. Salgado, Silvia Moscoso

**Affiliations:** Faculty of Labor Relations, University of Santiago de Compostela, Santiago de Compostela, Spain

**Keywords:** interrater reliability, supervisory performance ratings, appraisal purpose, scale type, range restriction, meta-analysis

## Abstract

**Objectives:** This reliability generalization study aimed to estimate the mean and variance of the interrater reliability coefficients (*r*_*yy*_) of supervisory ratings of overall, task, contextual, and positive job performance. The moderating effect of the appraisal purpose and the scale type was examined. It was hypothesized that the ratings collected for research purposes and multi-item scales have higher *r*_*yy*_. It was also examined whether *r*_*yy*_ was similar for the four performance dimensions.

**Method:** A database consisting of 224 independent samples was created and hierarchical sub-grouping meta-analyses were conducted.

**Results:** The appraisal purpose was a moderator of *r*_*yy*_ for the four performance dimensions. Scale type was a moderator of *r*_*yy*_ for overall and task performance collected for research purposes. The findings also suggest that supervisors seem to have less difficulty evaluating overall job performance than task, contextual, and positive performance. The best estimates of the observed *r*_*yy*_ for overall job performance are 0.61 for research-collected ratings and 0.45 for administrative-collected ratings.

**Conclusions:** (1) Appraisal purpose moderates *r*_*yy*_ and researchers and practitioners should be aware of its effects before collecting ratings or using empirically-derived interrater reliability distributions, (2) Scale type seems to moderate *r*_*yy*_ in the case of the ratings collected for research purposes, only, (3) overall job performance is more reliably rated than task, contextual, and positive performance. Implications for research and practice are discussed.

## Introduction

Job performance ratings are the most widely used criteria in Work and Organizational (W/O) Psychology (Landy and Rastegary, 1989; Borman, 1991; Woehr and Roch, 2012), and their importance as a dependent variable continues to be crucial for research (Vinchur, 2007; Van Iddekinge and Ployhart, 2008). Their frequent use runs parallel to the continued concerns of researchers regarding their reliability (Murphy and Cleveland, 1995; Murphy, 2008; LeBreton et al., 2014). For decades, a widespread concern about job performance ratings has been that they are affected by many errors, including halo and leniency, and that their reliability is low (Murphy and Cleveland, 1995; Campbell and Wiernik, 2015). For these reasons, it has been suggested many times that objective criteria (e.g., production records, work sample tests, sales quota) should be used instead of job performance ratings.

The concerns with the reliability of job performance ratings are not about its internal consistency (as estimated, for instance, by Cronbach's alpha and Spearman-Brown's formula) or its temporal stability (as estimated by a test-retest coefficient). The central distrust of some researchers is related to the interrater reliability of job performance ratings (Murphy and De Shon, 2000; LeBreton et al., 2003, 2014; Murphy, 2008). In other words, the skepticism is about the extent to which the scores given by a rater to a sample of incumbents correlate with the scores of a second rater to the same sample of incumbents, provided that the two raters have the same opportunity to observe the performance of incumbents, that they observe similar behaviors, and that they have similar job positions (e.g., the two raters are supervisors).

In validity studies, the rating sources of job performance can be the supervisors (e.g., Harris et al., 1995; Campbell and Wiernik, 2015), instructors (e.g., Berges et al., 2018), peers (e.g., Harris and Schaubroeck, 1988; Viswesvaran et al., 2002), and incumbents (e.g., Bang and Reio, 2017; Jyoti and Sharma, 2017; Haider et al., 2018; Rehman and Shahnawaz, 2018). This paper focuses on the interrater reliability of supervisory performance ratings as they are the most frequently used in validity studies and for performance appraisal purposes (Bernardin and Beatty, 1984; Landy and Rastegary, 1989; Viswesvaran et al., 2002; Campbell and Wiernik, 2015).

The importance of interrater reliability of supervisory performance ratings is related to the fact that, for years, researchers have considered this reliability coefficient to be the most relevant one for research (e.g., validation studies) and practice (e.g., administrative decisions). For example, Ghiselli et al. (1981), Guilford (1954), Guion (1998), Schmidt and Hunter (1996), Schmitt and Klimoski (1991), and Thorndike (1949), among others, have affirmed that for studies of supervisory performance ratings, the reliability coefficient of interest is an interrater coefficient. Critics of the use of supervisory ratings due to their low interrater reliability also seem to consider this reliability estimate as crucial (Murphy, 2008, 2014; LeBreton et al., 2014).

Primary studies (e.g., Rothstein, 1990) and meta-analyses (e.g., Viswesvaran et al., 1996) found the observed interrater reliability of supervisory ratings of overall job performance to be 0.52 on average (see also, Salgado et al., 2003, 2015a; Salgado and Tauriz, 2014) and, until very recently, this value seemed to be well-established. However, recently, several researchers have disputed the accuracy and legitimacy of this figure and criticized the use of 0.52 to correct validity coefficients for attenuation (e.g., LeBreton et al., 2003, 2014; Murphy, 2008). Other researchers have suggested that a provisional value of 0.80 would be a reasonable literature-based estimate of the reliability of supervisory performance ratings (e.g., Burke et al., 2014).

The main objective of this study was to investigate the interrater reliability of supervisory ratings of overall job performance and of three sub-dimensions: task performance, contextual performance, and positive work behavior (as opposed to counterproductive work behavior). We used meta-analytic methods to cumulate interrater reliabilities across studies and to estimate the degree of reliability generalization for the average interrater coefficient after the corrections for sampling error and range restriction. A second objective was to investigate the moderating effects of the appraisal purpose (i.e., administrative vs. research) on the interrater reliability of supervisory performance ratings. The third objective was to compare the interrater reliability for mono-item scales and multi-item scales of job performance measures. Finally, the fourth objective was to develop interrater reliability distributions that might be used in future studies (both, primary and meta-analytic studies) that include supervisory performance ratings.

## Research on Interrater Reliability of Supervisory Performance Ratings

One of the most highly cited studies on the interrater reliability of supervisory performance ratings was carried out by Rothstein (1990), who examined the effects of length of service on the interrater reliability coefficients in an extensive database consisting of 9,975 supervisors employed in a consortium of 79 companies. The length of service ranged from 0.5 months to over 240 months (i.e., over 20 years). Two raters rated each supervisor, one being the direct manager and the second a person well positioned to rate the supervisor. Length of service served as a subsidiary measure of the opportunity to observe the performance of the supervisor. Rothstein found that the frequency-based values of the observed interrater reliability were 0.48 for assessing duty ratings and 0.52 for assessing ability ratings, respectively. The average observed interrater reliabilities weighted by the sample size were 0.51 and 0.55 for duty ratings and ability ratings, respectively (based on the values reported by Rothstein, 1990). She also found that a non-linear quadratic function better described the relationship between the length of service and the interrater reliability coefficients.

Several characteristics of Rothstein's study should be mentioned here. First, the observed correlation between the interrater reliabilities for duty ratings and ability ratings was 0.88. Therefore, they are two empirically redundant measures (Schmidt et al., 2008). Second, range restriction affected the observed interrater reliability. Rothstein found that the correlation between the observed and the corrected reliabilities was 0.71 for duty ratings and 0.69 for ability ratings. She also found that, when the observed reliability coefficients were corrected for range restriction, the length of exposure (opportunity to observe) no longer contributed to the prediction of interrater reliability of duty ratings, and that its contribution was tiny for the interrater reliability of ability ratings. Finally, Rothstein's study (1990) is also relevant for the present reliability generalization study because she reported 37 individual coefficients of interrater reliability with their associated sample size and the variance of the ratings (which allows for the estimation of the μ-value for each study). When Rothstein's values are corrected for range restriction, the average interrater reliability is 0.64 for duty ratings and 0.69 for ability ratings.

Concerning the meta-analyses of the reliability of job performance ratings, the most comprehensive one was carried out by Viswesvaran et al. (1996). They examined the interrater reliability of the performance ratings made by supervisors and peers, and also examined the internal consistency and stability of job performance ratings. They also estimated the reliability for overall job performance and nine facets of performance, including quantity, quality, leadership, communication competence, administrative competence, interpersonal competence, effort, job knowledge, and compliance with or acceptance of authority. The most relevant finding of Viswesvaran et al.'s meta-analysis was to show that the observed interrater reliability (sample size weighted) was 0.52 (*K* = 40, *N* = 14,650) for supervisory ratings of overall job performance. The stability for supervisory performance ratings was 0.81, and the internal consistency (Cronbach's alpha) was 0.86.

Taking into account that the different reliability coefficients assign different sources of variance to measurement error (Schmidt et al., 2003), Viswesvaran et al.'s (1996) results clearly showed that the *intra-rater* reliability coefficients of supervisory performance ratings were high (i.e., over 0.80) but that the *interrater* reliability was low (i.e., 52). Therefore, Viswesvaran et al.'s (1996) findings showed that the primary source of error in supervisory performance ratings arises from the individual rater's idiosyncratic perceptions of job performance.

Two characteristics of Viswesvaran et al.'s (1996) meta-analysis must be mentioned. The first one is that their database included Rothstein's (1990) study, which represented about 75% of the total sample size of the meta-analysis. Thus, Rothstein's study was very determinative of the 0.52 value finally found. The second characteristic of Viswesvaran et al.'s (1996) meta-analysis is that all the studies included in their database were published studies conducted in the US and Canada. For this reason, new meta-analyses including published and unpublished studies carried out in other countries are essential. In an independent meta-analysis, Salgado et al. (2003) found that the observed interrater reliability of supervisory performance ratings was 0.52 in a sample of European studies (*K* = 19, *N* = 1,960). Therefore, the two independent meta-analyses arrived at the same observed interrater reliability coefficient (Salgado et al., 2016).

Despite the agreement between the independent meta-analyses mentioned above, some researchers disagree about the accuracy and legitimacy of the 0.52 estimate. For example, Murphy and De Shon (2000, p. 896) suggested that the interrater reliability found by Viswesvaran et al. (1996) may be a consequence of using contexts that encourage disagreement among raters, that promote substantial ratings inflation, and, consequently, produce range restriction. More recently, LeBreton et al. (2014; see also, LeBreton et al., 2003) disputed whether 0.52 is an accurate and reasonable estimate of interrater reliability of supervisory performance ratings. They also suggested that this estimate should not be used for correcting validity coefficients because “*the interpretation of a corrected coefficient when the criterion is measured using supervisory ratings has little scientific (or practical) value*” (LeBreton et al., 2014, p. 491). Finally, LeBreton et al. (2014, p. 497) stated that psychologists should “*take seriously the problems with the ratings and question whether interrater correlations between two supervisors provide the most accurate and reasonable estimates of reliability*.”

According to many classical psychometricians and W/O psychologists (e.g., Thorndike, 1949; Gulliksen, 1950; Guilford, 1954; Guion, 1965a, 1998; Nunnally, 1978; Ghiselli et al., 1981, among others), when the criterion measure is unreliable, what is of critical importance is that the sample size be increased in order to allow for sampling fluctuations and to get stability in the relative size of the validity coefficients.

### Appraisal Purpose: Administrative vs. Research

In the case of supervisory performance ratings, there are essential differences between ratings collected for administrative-purposes and research-purposes (Murphy and Cleveland, 1995). Many researchers have pointed out that performance assessment done for administrative purposes (e.g., promotions, compensation, prizes) is more complicated to evaluate because it may depend on group processes (e.g., rater-ratee similarity), and on contextual and organizational (e.g., tenure, reward system) factors (McDaniel et al., 1994; Viswesvaran et al., 1996, 2002; Tenopyr, 2002; Campbell and Wiernik, 2015). On this issue, Landy and Farr (1980) concluded that the administrative ratings were more lenient than the research ratings. Wherry and Barlett (1982) hypothesized that research ratings would be more accurate than administrative ratings, and empirical studies demonstrated that administrative ratings were significantly more lenient and exhibited more halo than did research ratings (Taylor and Wherry, 1951; Sharon and Barlett, 1969; Warmke and Billings, 1979; Wherry and Barlett, 1982; Zedeck and Cascio, 1982; Veres et al., 1983; Bretz et al., 1992). For example, Harris et al. (1995) found that the reliability of ratings for research purposes was larger than for administrative purposes. Besides, McDaniel et al. (1994) found evidence that the purpose of the job performance ratings (administrative vs. research) was a significant moderator of the criterion-oriented validity of employment interviews.

Murphy (2008), Murphy and Cleveland (1995), and Murphy and De Shon (2000) suggested that the accurate evaluation of subordinate performance is often a relatively minor concern of the rater, when the purpose of the appraisal is motivating subordinates, maintaining smooth interpersonal relations, and so on. Performance appraisal researchers typically assume that performance ratings are inflated, and rater effects generally lead to errors that are biased in the direction of leniency (Bretz et al., 1992; Cleveland and Murphy, 1992; Murphy and Cleveland, 1995; Jawahar and Williams, 1997; Murphy and De Shon, 2000). Research on performance appraisal also suggests that ratings are influenced by the goals pursued by raters (Murphy and Cleveland, 1995). Examples include using performance ratings to motivate subordinates or to build and maintain positive interpersonal relationships in the workgroup. There is evidence that raters' beliefs about the purpose of rating substantially affect the ratings they assign and there is also evidence that rater's attitudes toward the organization and the performance appraisal systems in use affect their ratings (Murphy and Cleveland, 1995; Tziner et al., 1998).

Therefore, research appears to support the hypothesis that ratings collected for research purposes are also more reliable than ratings collected for administrative purposes. Consequently, the aim of the collected ratings should be taken into account in the investigation of the interrater reliability of performance ratings as it may be considerably different for these two uses of supervisory performance ratings. However, no meta-analysis has estimated and compared, until now, the interrater reliability of research and administrative ratings. In line with the previous research and previous theoretical rationales, we state the following hypothesis:

***Hypothesis 1***: Interrater reliability is larger for ratings collected for research purposes than for administrative purposes.

### Single-Item Scales and Multiple-Item Scales of Job Performance

Assessments of overall job performance can be obtained either using (a) a single-mono-item scale, (b) using multiple-mono-item scales measuring more specific dimensions, (c) using multiple-multi-item scales, and (d) using multiple independent criteria that serve to create a composite measure (Guion, 1965a; Schmidt and Kaplan, 1971; Borman, 1991; Wilmot et al., 2014). The first two types of scales are the most frequently used in validation studies. For some administrative purposes (e.g., feedback, promotions), the multiple-multi-item scales and the composite performance measures are commonly used (Borman, 1991; Murphy and Cleveland, 1995).

Wilmot et al. (2014) affirmed that the 0.52 interrater reliability estimate found by Viswesvaran et al. (1996) is really the interrater reliability for single-scale measures of overall job performance. However, this may not be right. For example, the main contribution to Viswesvaran et al.'s (1996) meta-analysis was the study by Rothstein (1990), which used an instrument composed of 49 items, which tapped two performance constructs, i.e., duties and ability. Wilmot et al. (2014) suggested that, although single-scale and multi-scales tap the same construct, the multi-scale measures of job performance are likely to result in more reliable estimates. Therefore, according to Wilmot et al. (2014), a remedy for the low interrater reliability of overall performance ratings would be to use multi-scale measures and composite performance measures. This issue has scarcely been investigated, but there is some empirical evidence supporting Wilmot et al.'s point. For example, Salgado and Moscoso (1996) found that the interrater reliability was 0.45 for mono-item scales and 0.64 for multiple-item scales. Nevertheless, Viswesvaran et al. (1996) also found some evidence to the contrary. For example, they found that interrater reliability was larger for single scales assessing productivity (*r*_*yy*_ = 0.57), quality (*r*_*yy*_ = 0.63), administrative competence (*r*_*yy*_ = 0.58), effort (*r*_*yy*_ = 0.55), or compliance with authority (*r*_*yy*_ = 0.56), than for overall job performance (*r*_*yy*_ = 0.52). Consequently, this issue requires additional research.

Moreover, although larger internal consistency can be obtained if multiple homogenous scales are added up, as follows from the application of Spearman-Brown's prophecy formula, this is not necessarily the case for the interrater reliability. In other words, the idea that independent raters agree more among themselves on a ratee when a measure of overall job performance based on multiple scales is used than when a single scale of overall job performance is used does not follow obviously from the application of Spearman-Brown's formula. Of course, the larger the number of raters is, the larger the reliability coefficient is. However, this result is independent of whether the performance measure is a single scale or a multi-scale. It can be argued that, in some cases, the agreement can be smaller when multiple scales are used. For example, if the scales aim to measure badly-defined constructs or well-defined but rarely observed constructs (or the observer has difficulty in observing repeated behaviors), then it can be expected that the agreement will be smaller for the average of these scales than for an overall single-scale. The same result can be conjectured if the length of observation is short. Moreover, it is necessary to take into account whether the measure is formative or reflective (Edwards and Bagozzi, 2000; Salgado et al., 2015b). As noted by Bollen and Lennox (1991) and Edwards and Bagozzi (2000), the observed items and scales are correlated in the case of reflective constructs, but high correlations among indicators are not expected in connection with formative variables.

The compound measures as defined by Wilmot et al. (2014) seem to be formative variables and, consequently, the potentially larger interrater reliability associated with the use of these performance measures has not been demonstrated empirically. On the other hand, as reflective measures are more frequently used than formative measures in the most common developmental and validation studies, the following hypothesis is advanced:

***Hypothesis 2***: Interrater reliability will be greater for multi-item (multi-scale) measures than for single-item scales.

### Range Restriction in Interrater Reliability of Job Performance Ratings

It is widely admitted that reliability estimates (e.g., Alpha, test-retest, interrater coefficients) are affected by group variability so that the estimated reliability coefficients will be higher in more heterogeneous groups (e.g., Thorndike, 1949; Gulliksen, 1950; Guion, 1965a, 1998; Lord and Novick, 1969). Consequently, the reliability coefficients will tend to be smaller for a sample of job incumbents (i.e., typically a more homogenous group) than for the total population of job employees of a given organization. According to Feldt and Qualls, (1998, see also Hunter, 1983; Brennan, 1995), the basis for this reasoning is that the variance for the population of job incumbents equals the mean variance of the sub-samples plus the variance of the means. The importance of the effect of direct range restriction (DRR) on criterion reliability was stressed 40 years ago by Schmidt et al., (1976, p. 475) and by Callender and Osburn, (1980, p. 549), who mentioned that, in both local validation studies and validity generalization studies, the unrestricted criterion reliability must be used for correcting validity coefficients (even if assumed distributions of unrestricted criterion reliabilities were necessary) when range restriction on the predictor or predictors is direct. In the case of the indirect range restriction (IRR), as discussed by Schmidt and Hunter, (2015; see also Hunter and Schmidt, 2004; Hunter et al., 2006), the restricted values of reliability for both the criterion and the predictor must be used.

There are good reasons to suspect that restriction of range exists in the criterion scores (e.g., job performance ratings). Alexander et al. (1987) suggested that it was reasonable to assume that the criterion was restricted in range in many studies and some empirical evidence of DRR in performance ratings has been found (Rothstein, 1990; Bretz et al., 1992; Murphy and Cleveland, 1995). DRR may occur at the lower end of the criterion because the employees are self-selected or are dismissed and DRR may take place at the higher end because the employees are promoted or transferred or because they receive offers from other organizations that they subsequently accept (Sackett et al., 2002). DRR can also occur simultaneously at the lower and at the higher end because, for example, not all employees can be rated by two or more supervisors for different reasons, and because only a small number of employees can be rated by two raters, which is the most frequent case (Sackett et al., 2002). However, there are many other reasons. For example, it is commonly observed that raters do not use the full rating scale when they rate job incumbents (Murphy and Cleveland, 1995). Sometimes, raters cannot rate some incumbents because the period to observe the incumbent's work is short. Another reason is that sometimes the reliability coefficient is only calculated with an unrepresentative sub-sample of the population of employees (sometimes a very small sub-sample). Therefore, different mechanisms can operate to produce range restriction in the criterion (Sackett et al., 2002).

Furthermore, the effects of IRR and DRR over interrater reliability can be cumulative. For example, the effect of IRR is that only a sub-sample of applicants is finally hired and, the selection procedure (e.g., a GMA test) affects the reliability of supervisory performance ratings indirectly. In other words, the IRR operates on the applicant samples. On the other hand, the DRR operates on the employees (e.g., when the organization rates a selected sub-sample of the incumbents based on a variable, for instance, tenure). These two effects are independent, and therefore, their consequences for reliability can be cumulative.

Regardless of the range restriction mechanisms (i.e., direct or indirect), in all these cases, the observed distribution of the criterion scores would not reflect all the variability in the performance of incumbents. Consequently, the observed reliability estimated using the restricted distribution may not be best estimate of the true criterion reliability. Unfortunately, the effect of range restriction on the criterion reliability has received little attention. The study of Sackett et al. (2002) is an exception.

According to Murphy and De Shon (2000, p. 896; see also Murphy and Cleveland, 1995), the simpler explanation for the low interrater correlations is that performance ratings are typically collected in settings where range restriction is ubiquitous, especially when ratings are used to make administrative decisions about ratees (e.g., salary, promotion). Similarly, LeBreton et al. (2003) posited the hypothesis of restricted variance for explaining the low interrater reliabilities of supervisory performance ratings. They suggested that the low observed interrater reliability is an artifactual effect of range restriction. LeBreton et al. (2003) found some support for this hypothesis in peers and subordinates. Also, they recommend that the interrater reliability is corrected for attenuation due to range restriction.

In an important study, Sackett et al. (2002) examined the effects of range restriction on the interrater reliability using three simulated scenarios, including DRR and IRR. They found that the consequences of the range restriction on the criterion reliability varied considerably depending upon the specific mechanism producing range restriction and the degree of range restriction presented, as indexed by the selection ratio. Sackett et al.'s (2002) findings showed that DRR produced the largest effect and that it can result in substantial underestimation of criterion reliability. Consequently, if the uncorrected reliability estimates are used for correcting validity coefficients, there is a possibility of substantial overestimation of the validity (Sackett et al., 2002). On the contrary, if reliability estimates are corrected for range restriction before their use in correcting validity coefficients for attenuation, true validity will be estimated accurately (Callender and Osburn, 1980; Sackett et al., 2002).

Viswesvaran et al. (2014) pointed out that range restriction, together with sampling error, and other statistical artifacts (e.g., imperfect construct measurement) have effects on the observed interrater reliabilities of supervisory performance ratings, especially if the coefficients are from single studies. Consequently, meta-analytic observed values provide more accurate estimates than the coefficients of single studies, because of the sampling error reduction. Furthermore, if the observed interrater reliabilities are corrected for range restriction, these reliabilities would be even less biased and more precise estimates.

In summary, the third explanation for low interrater correlations is that the samples of job incumbents, which served to estimate the interrater reliability, exhibited less variability than the respective job incumbent populations and, therefore, a DRR mechanism operated to attenuate the true reliability. Furthermore, the observed interrater reliability estimates can also be indirectly restricted due to the effects of the personnel selection procedure (e.g., a cognitive test) on the criterion scores. The explanation based on range restriction is independent of the previous one based on the appraisal purpose. However, no previous meta-analyses of the interrater reliability of supervisory performance ratings (Salgado and Moscoso, 1996; Viswesvaran et al., 1996; e.g., Salgado et al., 2003) estimated the degree of range restriction in the ratings. Thus, a goal of this reliability generalization study is to examine the effects of RR on interrater reliability magnitude and variability on interrater reliability estimates.

### Job Performance Dimensions

Viswesvaran et al. (1996) pointed out that in research literature the same facets of job performance can be referred to by different labels. For this reason, they suggested that theoretical considerations should guide the grouping of the different facet labels. In this research, we have used four performance dimensions (clusters): Overall job performance, task performance, contextual (citizenship) performance, and positive organizational performance (i.e., the positive pole of counterproductive work behaviors). The definition of these performance categories appears in Table 1. Carpenter and Berry (2017), Harari et al. (2016), and Hoffman et al. (2007) reviewed the literature on the definition and measurement of these three performance dimensions. The meta-analysis of Hoffman et al. (2007) reported a correlation between task performance and contextual (citizenship) performance of 0.74, and the meta-analysis of Carpenter and Berry (2017) reported a correlation of −0.22 between contextual performance and counterproductive work behaviors and a correlation of 0.06 between task performance and counterproductive work behaviors. Therefore, these meta-analytic findings support the contention that the three dimensions, although related, are empirically different. For research and practical motives, it is relevant to estimate their specific interrater reliability.

**Table 1:** Definitions of overall job performance, and the sub-dimensions.

***Overall job performance*:** Ratings on statements (or ranking of individuals on statements) referring to overall performance, overall effectiveness, overall job performance, overall work reputation, or the sum of all individual dimensions rated” (Viswesvaran et al., 1996, p. 561).
***Task Performance*:** Ratings of behaviors, abilities, and competencies referring to the production of a good or the provision of a service.
***Contextual (Citizenship) performance*:** ratings of behaviors that contribute to the goals of the organization contributing to its social and psychological environment.
***Positive performance (as opposite to counterproductive work behaviors)*:** Ratings of voluntary behaviors that contribute to the well-being of the organization (e.g., maintaining personal discipline, avoidance of dangerous-destructive-hazardous behaviors, personal compliance).

We do not advance any hypothesis about potential differences among the interrater reliability of the performance dimensions as the current theoretical and empirical literature does not provide a sound basis to speculate about potential differences and to posit hypotheses might be premature. For example, Borman (1979) and (Wohlers and London, 1989) suggested that some performance dimensions (e.g., administrative skills, communication skills and leadership) can be easier to assess than others (e.g., quality and productivity). To this regard, Viswesvaran et al. (1996) found that, although there was some variability in the average interrater reliability across the 10 dimensions included in their meta-analysis, the 80% credibility intervals and the 95% confidence interval overlapped (Viswevaran et al. reported 80% confidence intervals but the 95% confidence interval can be calculated with the estimates they reported). Nevertheless, the comparison of the interrater reliability of the job performance dimensions may contribute to a better understanding of the appraisal processes by supervisors. Thus, an additional purpose of this reliability generalization study is to estimate the interrater reliability of three dimensions of job performance and overall job performance.

## Method

### Literature Search

Using four strategies, we have searched for studies that reported interrater reliability coefficients either for overall job performance or job performance sub-dimensions and facets. The first strategy was to examine all the articles included in the database of Viswesvaran et al. (1996) and to retain the articles that reported interrater reliability coefficients. This strategy provided about 42 documents (25% of the references to studies finally included in the database) which provided 78 interrater coefficients. The second strategy was to conduct electronic searches using the following databases and meta-databases: PsycLit, Google, Scholar-Google, ERIC, Elsevier, Sage, Wiley, Academy of Management, Springer, and EBSCO. We used the following keywords: “interrater reliability” in combination with “job performance,” “task performance,” “contextual performance,” “citizenship performance,” “performance ratings,” “work performance,” “performance appraisal,” “performance evaluation,” and “performance assessment.” With this strategy we obtained 1,387 references and we examined the full content of each document. We excluded the majority of the documents mainly because (a) they did not provide an interrater coefficient of job performance, (b) they provided an interrater coefficient but for the predictors and not for job performance; (c) they provided an interrater coefficient of job performance but the coefficient was obtained correlating the ratings of peers or subordinates; (d) some documents provided Cohen's Kappa and other coefficients of agreement but they did not calculate a correlation coefficient (e.g., Pearson; intraclass); (e) some valid documents were excluded because they were previously obtained from Viswesvaran et al.'s (1996) list of references. This strategy provided 96 usable documents (117 interrater coefficients).

The third strategy was to examine all issues (starting from the first issue) of some relevant journals not included in Viswesvaran et al.'s list of journals. We examined the following journals: International Journal of Selection and Assessment, the Journal of Work and Organizational Psychology, the European Journal of Work and Organizational Psychology, Applied Psychology, and Educational and Psychological Measurement. Moreover, we also examined the issues from January 1994 to December 2017 of the journals listed by Viswesvaran et al. (1996). This strategy provided 25 documents (15%) and 27 interrater coefficients. Finally, the fourth strategy was to contact fifteen international researchers from USA, United Kingdom, Germany, Italy, The Netherlands, Belgium, Spain, and France to obtain previously unidentified papers. We obtained 6 documents (with 14 interrater coefficients) from our colleagues.

### Inclusion Criteria and Decision Rules

As the main purpose of this reliability generalization study was to determine the interrater reliability of the supervisory ratings of overall, task, contextual, and positive performance, only studies that reported an estimate of this reliability form were included in the analysis. Furthermore, in the final database, we included only studies reporting actual supervisory-job-performance ratings with real incumbents. In other words, we did not consider experimental studies, studies with simulated people, and studies with students. We also excluded studies reporting interrater reliability estimates of interview ratings, interrater reliability of assessment center exercises, and interrater reliability estimates of performance in simulated exercises. As we were interested in supervisory interrater reliability, the estimates had to be calculated using supervisory ratings and we did not consider interrater coefficients obtained from peers, subordinates, and observers. Also, we did not consider interrater coefficients obtained from a single supervisor supplemented by a peer or an observer. When studies reported a range of numbers of incumbents, we coded the smaller number to provide a more conservative estimate (e.g., Dunnette and Motowidlo, 1976; Campbell, 1986; Blickle et al., 2008). When an article or document reported data from two or more independent samples of participants they were entered into the meta-analysis as separate interrater reliability estimates (e.g., Dunnette and Motowidlo, 1976; Campbell, 1986). When a study reported interrater reliability estimates for the same sample obtained on different occasions, the most recent estimate served as data source for that sample (e.g., Gunderson and Ryman, 1971). When a study used two performance measures for the same sample at the same time (e.g., ranking order plus Likert scale; graphic ratings plus paired comparison ratings), the average of the interrater reliabilities was enter as the data source (e.g., Mandell and Adkins, 1946; Buel and Bachner, 1961; Thompson and Thompson, 1985). Handyside and Duncan (1954) reported an interrater coefficient that had been corrected for range restriction and we transformed it back to the observed interrater reliability. We also excluded 11 studies because the same coefficients had been reported in another paper included in the dataset.

Per the Meta-Analysis Reporting Standards (MARS) specified in the Publication Manual of the American Psychological Association (2010; available at https://apastyle.apa.org/manual/related/JARS-MARS.pdf), the Preferred Reporting Items for Systematic Reviews and Meta-analysis (PRISMA), and the checklist of meta-analysis of Aytug et al. (2012), we have included as Supplementary Material a file containing the following information from each study for each job performance category: (a) study source, (b) *r*_*yy*_, (c) N, (d) type of scale (mono vs. multi-item), (e) purpose (administrative vs. research), and (f) range restriction value.

### Summary of Interrater Reliability Data Set

The final number of independent documents (i.e., articles, technical reports, presentations, doctoral dissertations, and unpublished manuscripts) we were able to use in this meta-analysis was 169, of which 108 (65.7%) were published studies and 58 (34.3%) were unpublished studies. These documents provided 236 independent samples/studies in which supervisory interrater reliability estimates were reported. The published studies provided 160 independent samples (68%) and the unpublished studies provided 76 samples (32%). The total sample size was 43,203 individuals. The oldest study was published in 1933 (Farmer, 1933) and the most recent was published in 2017 (Lado and Alonso, 2017). Therefore, the time period covered by this meta-analysis was 84 years. As for the language of the documents, 159 (94%) were in English, 7 (4.1%) in Spanish, 3 (1.8) in French, and 1(0.1%) in German. The list of the documents appears in the section of references.

### Inter-coder Agreement

The two authors of this reliability generalization study, both with experience conducting meta-analyses, with a number of published meta-analytic studies in top-tier journals (e.g., Journal of Applied Psychology; Personnel Psychology; Journal of Occupational and Organizational Psychology; European Journal of Work and Organizational Psychology), with research and teaching experience in personnel selection, both with doctorate degrees in Work and Organizational Psychology, coded independently all interrater reliability studies. For each study, we compared eight data points: (a) sample size; (b) interrater reliability; (c) job performance category; (d) administrative vs. research purpose; (e) mono vs. multiple-item scales; (f) range restriction value; (g) published vs. unpublished study; (h) study year. To establish the level of inter-coder agreement, we identified the number of data points and the number of disagreements. For this analysis, there were 2,218 data points, with 2,085 agreements and 133 disagreements, yielding a 94% level of agreement. Disagreements were resolved by referring back to the studies and discussion between the two authors, until consensus was reached. This level of agreement is similar to the inter-coder agreement found in validity generalization studies (Whetzel and McDaniel, 1988; Whetzel et al., 2014).

### Method for Reliability Generalization of Interrater Coefficients of Job Performance

In this meta-analysis, one interrater reliability coefficient was used per sample for each performance dimension-moderator condition. In other words, we fixed the contribution of each study at a single reliability coefficient for overall job performance and for each performance category.

One-hundred-and-eight studies contained conceptual replications (i.e., interrater reliability coefficients of two or more facets of the same performance dimension were obtained in the same sample). In 45 cases, we formed the linear composites when the study reported the intercorrelations among the scales. Linear composites with unit weights for the components provide more construct valid and precise estimates than the use of the average correlation or the use of all correlations as separate data points (Nunnally, 1978, p. 166–168; Viswesvaran et al., 1996, 2002; Schmidt and Hunter, 2015, p. 457–463). Composite interrater correlations represent the interrater reliability that would have been observed had the scales been summed (Connelly and Ones, 2010). In the case of the 63 studies that did not contain the correlations among the facets or scales, we used the average correlation.

When a study reported the interrater reliability of several facets together with the interrater reliability of overall job performance, we used this last estimate as the interrater reliability for overall job performance. We used an identical procedure when the facets were for task performance, contextual performance, and positive performance and an estimate of these dimensions was also provided.

When a study reported interrater reliability estimates for overall job performance and the dimensions of task, contextual, and positive performance, each specific coefficient was assigned to the group of coefficients of each dimension. In this way, although such a study can provide several coefficients, the independence of the coefficients is not violated within the same performance dimension. In other words, the interrater coefficients of overall job performance were not collapsed into the coefficients of task, contextual, and positive performance, nor were the coefficients of these dimensions grouped together.

The vast majority of the studies (94.5%) used two raters for estimating the interrater reliability, but 12 (5.5%) studies reported that three or more raters had been used to assess the interrater reliability. In other words, the reliability estimate can be different across the studies because of the number of raters of the performance measurement. In this case, we have followed the methodological strategy used by Viswesvaran et al. (1996) and reduced all interrater estimates to that of one rater using the Spearman-Brown prophecy formula.

The reliability generalization method used here can be divided into two parts. The first part is a bare-bones meta-analysis and it provides the estimates of the observed (uncorrected) interrater reliabilities. This part reports the following data: (a) the total number of interrater coefficients (*K*), the total sample size (*N*), the weighted-sample average observed interrater reliability (*r*_*yy*_), the weighted-sample observed variance of the observed interrater reliability (*S*_*yy*_^2^), the standard deviation of the observed interrater reliability (*SD*_*yy*_), and the sampling error variance of the observed interrater reliability (SEV). These estimates can be directly compared with Viswesvaran et al.'s (1996) estimates. We conducted the bare-bones meta-analysis with the meta-analytic software of Schmidt and Le (2014). In addition, we programmed the formulas in the spreadsheet Excel. The results of both Excel and Schmidt and Le's software were identical.

The second part contains a difference with respect to the method typically used in validity generalization research. In these last studies, the correction for range restriction is for the restriction which took place in the predictor scores. Thus, the formulas named as Thorndike's (1949) Case II and Case III, and the more recently derived formula of IRR by Hunter et al. (2006) are used to obtain unrestricted validity coefficients. In the present case, the restriction is on the criterion scores, and the correction for range restriction requires a different formula.

The criterion scores can be restricted for two main reasons. First, the range of the criterion scores can be *indirectly* restricted due to the effect of another variable (e.g., a predictor) that can reduce the variance of the performance in the incumbent samples. For example, if only the top 10% of applicants are hired or because the organization excluded some employees based on demographic variables (e.g., the ratings are only available for younger and less experienced employees), indirect range restriction affected the performance measures. In personnel selection, this is the most common situation (Thorndike, 1949; Hunter et al., 2006; Fife et al., 2012; Schmidt and Hunter, 2015). In fact, all job performance measures are indirectly restricted in range. Therefore, the direct restriction of the range of a predictor always produces indirect restriction in the range of the performance measure.

Second, the range of the criterion scores can be *directly* restricted when the performance measures are used for making personnel decisions and a sub-sample of employees is rated rather than the totality of the employees available in an occupation or an occupational type (Sackett et al., 2002). Direct range restriction can occur, for instance, when performance is used for promotions and when performance measures are used to transfer those who could not do the job (Guion, 1965a). In this last case, the indirect range restriction produced during the selection process is supplemented by the direct range restriction when the performance measure is used for personnel decisions such as promotions or transfers.

In this meta-analysis, we used the formula of Kelley-Otis (Kelley, 1921; Otis, 1922) to correct interrater reliability estimates for indirect range restriction (for this formula see also, Thorndike, 1949; Gulliksen, 1950; Guilford, 1954; Guion, 1965a; Lord and Novick, 1969; Nunnally, 1978; Schmidt and Hunter, 2015). The formula is as follows:

$$\begin{array}{l} R_{yy}=1-\left[u^{2}\left(1-r_{yy}\right)\right] \end{array}$$

where,

*R*_*yy*_ = corrected criterion reliability

*r*_*yy*_ = observed (restricted) criterion reliability

*u* = value of range restriction (restricted sd/unrestricted SD).

Fife et al. (2012) carried out an important study in which they reviewed the formulas required for correcting reliability estimates for range restriction. They found that the Kelley-Otis formula worked very efficiently under indirect range restriction, even when the selection ratio was as severe as 0.20. They also analyzed the assumptions and conditions of the use of this formula. This formula is based on two assumptions: (1) true scores and errors remain independent after range restriction; and (2) range restriction does not affect the size of the residual variance. According to Fife et al. (2012), in the case of the variables under indirect range restriction, like job performance measures, assumptions 1 and 2 are not problematic and the Kelley-Otis formula approximates unrestricted reliability accurately up to selection ratios of less than 0.2, when the formula produces overestimations of the reliability. Fife et al. (2012) also found that the standard error of the corrected estimates was very acceptable until restriction became severe (selection ratios less than 0.2), with bias smaller than 1%. Based on their simulation study, Fife et al. (2012) recommended correcting reliability coefficients under indirect range restriction to estimate the population interrater reliability.

The main difficulty in using this formula is to obtain the *u*-value, i.e., the coefficient of homogeneity of the criterion. We obtained the *u*-values from three sources. First, in the current database, 190 studies (77.88%) did not show evidence of range restriction, therefore we used a coefficient of *u* = 1 in these cases. Second, seven studies (2.04%) reported *u* values for the criterion. Third, 10 studies (4.09%) showed some degree of restriction as the effective sample of employees used for estimating the reliability was smaller than the total number of employees in the occupation (e.g., because of the use of an age criterion or the length of experience for rating the incumbent). Regarding this point, the effective sample of employees refers to the number of employees used to estimate interrater reliability. Fortunately, if both the effective sample of employees and the total sample size are known, then it is possible to obtain the selection ratio, and the *u*-value can be estimated from this value. Kelley, (1947; see also; Schmidt et al., 1976; Sands et al., 1978) derived the following formula for obtaining *u*-value from the selection ratio:

$$\begin{array}{l} u^{2}=1+\frac{z^{*}y}{\phi }-{\left(\frac{y}{\phi }\right)}^{2} \end{array}$$

where

ϕ = selection ratio,

*z* = standard normal deviate corresponding to the selection ratio,

*y* = ordinate on the standard normal curve that corresponds to *z*.

This formula assumes that the scores are normally distributed, and large separations of normality can result in severe underestimation of *u* value, which, in turn, may produce larger estimates of the correlation coefficients corrected for range restriction. In other words, reliability can appear as larger than it really is. Kelley (1947), Blixt and Shama (1986), and Feldt and Qualls (1998) examined the normality distribution assumption and found that the formula functions very efficiently in the most common range of *u* values (e.g., selection ratio > 0.60).

Furthermore, the statistical normality of the distributions of job performance ratings has been examined several times. For example, Kaiser et al. (2002, cited by LeBreton et al., 2003) found that job performance was normally distributed in a large sample of executives of a global technology firm. More recently, Beck et al. (2014), using 117 validation studies for different jobs (*N* = 21,945), found that a normal distribution provided a better fit to the supervisor ratings than a skewed distribution in all of the 117 studies. Also, they did not find evidence that job performance can be expected to be highly skewed. Also, Díaz-Vilela et al. (2015) found a normal distribution of administrative ratings of task and contextual performance in a sample of Spanish public servants. Based on these findings, it can be reasonably assumed that supervisory performance ratings are normally distributed and, therefore, that the reliability correction formula and the selection ratio formula can be applied in the present study. An additional test of the formula has been done with the data reported by Campion et al. (1988). They reported the selection ratio, the SD of applicants, and the SD of incumbents in a validation study. The *u*-value obtained dividing *SD*_*s*_ by *SD*_ρ_ was 0.65 and the *u*-value obtained with the selection ratio formula was 0.656. Consequently, the formula appears to work very efficiently, at least in the case of the ratings collected for research purposes.

The statistical normality of the distribution of job performance in the case of the ratings collected for administrative purposes has been less examined, although, as mentioned before, larger skew estimates can be expected. Consequently, this method of estimating *u-*values was not used in the ratings collected for administrative purposes, as it can overestimate the real restriction. The potential effects of this last decision are practically irrelevant for the final estimates, as only two studies with ratings collected for administrative purposes showed a small degree of restriction (average selection = 0.78; total sample = 198).

Third, Rothstein's (1990) study reported 37 interrater coefficients (15.61% of total coefficients), together with the respective sample size and the between subjects observed variance of duty performance ratings and ability performance ratings. This information is very useful because it permits the estimation of the population (large sample) variance. The interrater coefficients reported by Rothstein are mathematically equivalent to Fisher's (1928) intraclass correlation coefficients (ICC) (McNemar, 1962). The formula for this coefficient is:

$$\begin{array}{l} ICC\left(1,1\right)=\frac{S_{between}^{2}-S_{within}^{2}}{S_{between}^{2}+S_{within}^{2}} \end{array}$$

So the variance within studies is:

$$\begin{array}{l} S_{within}^{2}\;=\;\frac{S_{between}^{2}*\left(1-r_{yy}\right)}{\left(1+r_{yy}\right)} \end{array}$$

Consequently, using the interrater reliability and the between variance, it is possible to obtain the within-subject variance for each study. The population (total) variance is the sum of the two average variances, i.e., *S*^2^_*total*_ = *S*^2^_*within*_ + *S*^2^_*between*_. The square root of *S*^2^_*total*_ is the standard deviation of the population (*SD*_*p*)_. Now, dividing the SD of every single study by the *SD*_*p*_, the *u* value of each study can be estimated.

Therefore, there were three potential sources to obtain the *u*-values for the current dataset. The first source was the ratio between the *SD*_*s*_ and the *SD*_*p*_ and it provided seven coefficients (2.87%) The second source was based on the selection ratio and it provided 10 coefficients (4. 09%). The third source relied on the ICC formula and it provided 37 (15.16%) coefficients for Rothstein's (1990) study. In addition, 190 studies (77.88%) showed no evidence of range restriction. We have used the appropriate source in this reliability generalization study, depending on the information provided in the primary studies. Table 2 reports the distribution of *u* values for the different criterion sets. The next step was to apply the Kelley-Otis formula to obtain the estimate of the corrected reliability.

**Table 2:** Range restriction distributions of reliability coefficients of performance ratings.

**Variable**	**μ-mean**	***SD***
**Overall job performance (OJR)**	0.925	0.139
OJR-administrative	0.959	0.095
OJR-administrative-multi-item	0.962	0.097
OJR-research	0.922	0.141
OJR-research-multi-item	0.898	0.153
OJR-research-mono-item	0.981	0.083
**Task performance (TP)**	0.883	0.145
TP-administrative	1.0	0
TP-administrative-multi-item	1.0	0
TP-research	0.875	0.147
TP-research-multi-item	0.858	0.146
TP-research-mono-item	0.950	0.143
**Contextual job performance (CP)**	0.950	0.140
CP-administrative	1.0	0.000
CP-research	0.941	0.150
CP-research-multi-item	0.928	0.160
CP-research-mono-item	0.964	0.134
**Positive job performance (PP)**	0.970	0.095
PP-Research	1.0	0

Another relevant characteristic of this reliability generalization study is that the true (corrected) variance was estimated. In the present research, sampling error and indirect range restriction were the sources of artifactual variance considered. As the interrater reliability is a correlation coefficient, the formula for sampling error variance is the same for the present reliability generalization study and the validity generalization studies. Therefore, the next step was to estimate the sampling error variance in the corrected reliabilities. This was done using the formula derived by Bobko and Rieck, (1980; see also Schmidt and Hunter, 2015). This formula is:

$$\begin{array}{l} SE_{Ryy}=\frac{U}{{\left[1+r_{yy}^{2}\left(U^{2}-1\right)\right]}^{3/2}}SE_{ryy} \end{array}$$

Once the interrater reliabilities were corrected for range restriction and their variance estimated, the following averages were computed (using the study sample size for weighting the individual studies): (a) the average weighted-sample size corrected interrater reliability; (b) the variance of the corrected interrater reliabilities, and (c) the average sampling error variance of the corrected reliabilities, (d) the true variance of the corrected interrater reliabilities. Also, the increase in reliability due to the correction for range restriction and the percentage increase were calculated. Finally, the 80% credibility interval and the 95% confidence interval were calculated.

For the second part of this meta-analysis (i.e., the interrater reliability estimates corrected for range restriction), we programed the required formulas in Excel as, to the best of our knowledge, the current meta-analytic software available (e.g., Schmidt and Le's program, Comprehensive Meta-analysis; Meta-Win, and so on), does not include formulas to correct the reliability coefficients for range restriction and to obtain the corrected variance, corrected standard deviation, the standard error to the interrater reliability estimates, the 80% credibility interval, and the 95% confidence interval.

### Moderator Analysis

In this reliability generalization study, we examined two potential moderators of the interrater reliability of performance ratings. There are two related issues that must be considered concerning the analysis of moderators. The first one is if the analysis should be conducted one by one for each moderator variable or if a fully hierarchical meta-analysis should be conducted in order to isolate the true effects of the moderators. The second issue is about the approach to be used to determine if the observed variability in the interrater reliability is due to moderator variables.

According to Schmidt and Hunter, (2015, p. 383), when two or more moderators are suggested, it is correct to analyze the moderators separately if the researcher can correctly assume that (1) the moderator variables are independent and (2) the moderator variables are additive in their effects. Otherwise, a fully hierarchical meta-analysis should be conducted in order to detect the true influences of the moderators on the interrater reliability estimates and the interaction of the moderators. The typical subgrouping approach can be deceptive if moderators are correlated because the potential influence of a moderator can be a product of the real influence of another moderator with which it correlates. We know of no theoretical reason to assume that the ratings purpose and the scale type must correlate. However, in applied settings, it is more frequent that the ratings for administrative purposes are collected with multi-item scales. Therefore, when possible, we conducted separate meta-analyses for the combinations of the moderators for each performance category: purpose of ratings (administrative vs. research ratings) and type of scale (mono-item vs. multi-item). We were not able to analyze the moderating influences of the combination of administrative ratings and type of scales for some performance categories as the number of studies was very small (three or less) and the total sample size was also very small (<200 individuals).

Concerning the identification of moderators, there is wide consensus on the process used to analyze moderators in the meta-analysis of effect sizes, in general, and in validity generalization studies, in particular. Three common practices are to apply Q tests or similar, to use the 75% rule, and to use the 80% credibility interval overlapping zero (Aguinis et al., 2008; Schmidt and Hunter, 2015). These three approaches are not appropriate for reliability generalization studies because of: (a) the low statistical power (Q tests), (b) the subjectivity of the 75% rule, and (c) the practical impossibility of a reliability coefficient equal to zero, which renders useless the criterion of the 80% credibility interval overlapping zero. Other limitations of these approaches have been reviewed by Aguinis and Pierce (1998); Aguinis et al. (2008) and Schmidt and Hunter (2015).

In the case of the reliability generalization studies an approach posited by Schmidt and his colleagues (Viswesvaran et al., 2002; Hwang and Schmidt, 2012) can be used to determine if the observed variability is large enough to suggest the existence of moderators. The approach consists of a three-step process. First, it is necessary to observe if the width of the 80% credibility interval is large, which means that a great deal of variability remains unexplained by artifactual errors. Hwang and Schmidt (2012) suggested that an 80% credibility interval of 0.38 is large enough to suggest that a moderator analysis may be in order and Koslowsky and Sagie (1993) found that credibility intervals as small as 0.11 can indicate the presence of moderators. Second, if the credibility interval is large, the sample of studies must be broken into subgroups and separate meta-analyses conducted in accordance with the theory that suggests the existence of moderators. Third, a confidence interval (e.g., 90%) must be constructed around the mean corrected interrater reliability, using the standard error of the population. Sometimes, credibility intervals and confidence intervals are confused, but they provide different information. The credibility interval suggests the percentage of the population estimates (e.g., interrater reliability coefficients) that falls into the range of the interval. They are constructed using the standard deviation of the population. Confidence intervals are constructed around the mean corrected estimate using the standard error and they provide a measure of the error in the estimate of the population mean (Whitener, 1990; Hwang and Schmidt, 2012; Schmidt and Hunter, 2015). If the 95% subgroup confidence intervals do not substantially overlap (e.g., 25% or smaller overlapping) or, alternatively, if the lower limit of the 90% confidence interval of the difference of means is different from zero, then the moderator suggested by the theory is supported. The lower bound of the 90% confidence interval is the 95% confidence value.

We used this three-step approach in this reliability generalization study to test if the empirical evidence supported the existence of the two moderators suggested by theory, i.e., rating purpose and scale type.

### Publication Bias

Rothstein et al. (2005) defined publication bias as “the term for what occurs whenever the research that appears in the published literature is systematically unrepresentative of the population of completed studies” (p. 1). A critical issue of all meta-analyses is the potential bias due to the availability of one kind of studies only (e.g., published studies; significant studies). Although we have made a significant effort to include as many published and unpublished studies as possible, the average estimates could be affected by some kind of publication bias. Several methods to examine publication bias are currently available for meta-analyses of effect sizes, but they have not been checked for the case of reliability estimates. In addition, some of the methods are of questionable efficacy (see Becker, 2005; Kepes et al., 2012; Schmidt and Hunter, 2015, for reviews of the publication bias methods). Following the recommendations of Borenstein (2005), Kepes et al. (2012), and Schmidt and Hunter (2015), this reliability generalization review incorporates four methods of detecting potential publication bias: (a) subgroup comparison of published and unpublished studies; (b) Pearson correlation between the publication year and the interrater reliability size; (c) Orwin's failsafe N; and (d) subgrouping cumulative meta-analysis with a forest plot of the average estimates of the interrater reliability for the four categories of job performance ratings. A particularly relevant point in publication bias analysis is that the moderator variables must be taken into account when the particular method is applied (Schmidt and Hunter, 2015). In this study, we considered the publication source (published vs. unpublished studies), the rating purporse (administrative vs. ressearch ragints), and the study year as potential sources of publication bias.

## Results

### Reliability Generalization Results

Table 3 presents the bare-bones meta-analytic results for the interrater reliability of the four job performance categories and Table 4 reports the results for the interrater reliability estimates corrected for indirect range restriction.

**Table 3:** Bare-bones interrater-reliability generalization results for supervisory performance ratings.

**Purpose**	**K**	**N**	***r_***yy***_***	***r_***yy*_*f***_***	***${\text{Sr}}_{yy}^{2}$***	***SDr_***yy***_***	**SEV**	**SE*_***r***_***	**%VE**	**95%CI*_***ryy***_***
**Overall job performance ratings (OJR)**	219	41773	0.56	0.63	0.018	0.133	0.002	0.009	14.00	0.54/0.58
OJR-administrative	18	13632	0.45	0.55	0.014	0.120	0.001	0.028	5.82	0.40/0.41
OJR-administrative-multi-item	14	13272	0.45	0.52	0.014	0.118	0.001	0.032	4.84	0.38/0.51
OJR-research	201	28141	0.61	0.64	0.011	0.106	0.003	0.007	24.89	0.60/0.63
OJR-research-multi-item	145	23190	0.61	0.62	0.011	0.105	0.002	0.009	22.26	0.59/0.63
OJR-research-mono-item	56	4951	0.62	0.67	0.012	0.111	0.004	0.015	35.67	0.59/0.65
**Task performance (TP)**	94	28801	0.47	0.52	0.010	0.100	0.002	0.010	20.80	0.45/0.49
TP-administrative	6	10179	0.38	0.46	0.004	0.061	0.000	0.025	11.36	0.34/0.43
TP-administrative- multi-item	5	10120	0.34	0.42	0.003	0.059	0.000	0.026	10.45	0.33/0.43
TP-research	88	18622	0.52	0.52	0.007	0.085	0.002	0.009	35.33	0.50/0.53
TP-research-multi-item	71	14791	0.52	0.51	0.004	0.064	0.003	0.008	62.87	0.50/0.53
TP-research-mono-item	17	3831	0.51	0.59	0.016	0.125	0.003	0.030	15.84	0.45/0.56
**Contextual performance (CP)**	43	15721	0.43	0.54	0.021	0.144	0.002	0.022	8.78	0.38/0.47
CP-administrative	6	10184	0.36	0.46	0.006	0.076	0.000	0.031	7.87	0.30/0.42
CP-research	37	5537	0.56	0.56	0.022	0.150	0.003	0.025	14.11	0.51/0.61
CP-research-multi-item	23	2770	0.56	0.54	0.011	0.106	0.004	0.022	34.67	0.51/0.60
CP-research-mono-item	14	2767	0.56	0.58	0.034	0.183	0.002	0.049	7.15	0.46/0.65
**Positive performance (PP)**	10	2015	0.48	0.51	0.026	0.162	0.003	0.051	11.17	0.38/0.58
PP-research	8	1856	0.48	0.51	0.028	0.167	0.002	0.059	9.27	0.36/0.59

*K, number of interrater coefficients; N, sample size; r_yy_, weighted-sample average observed interrater reliability; r_yy_f_, frequency-weighted average observed interrater reliability; ${\text{Sr}}_{\text{yy}}^{2}$, observed variance; SDr_yy_, observed standard deviation; SEV, sampling error variance; %VE, percentage of explained variance; SE_r_, standard error of the observed interrater reliability; 95%CI_ryy_, 95% credibility interval of the observed interrater reliability; OJR, overall job performance ratings; TP, task performance ratings; CP, contextual performance ratings; PP, positive performance ratings*.

**Table 4:** Range-restriction corrected-reliability generalization results for supervisory performance ratings.

**Category**	***R_***YY***_***	***VAR_***RYY***_***	***SD_***RYY***_***	**SE*_***RYY***_***	**80%CrI*_***RYY***_***	**95%CI*_***RYY***_***	**Δ**	**%Δ**
**Overall job performance ratings (OJP)**	0.61	0.026	0.162	0.011	0.40/0.82	0.59/0.63	0.05	8.93
OJR-administrative	0.45	0.015	0.121	0.029	0.30/0.61	0.40/0.51	0	0.00
OJR-administrative-multi-item	0.45	0.014	0.119	0.031	0.30/0.60	0.39/0.51	0	0.00
OJR-research	0.69	0.015	0.121	0.008	0.54/0.84	0.67/0.71	0.08	13.11
OJR-research-multi-item	0.70	0.015	0.121	0.010	0.55/0.86	0.68/0.72	0.09	14.75
OJR-research-mono-item	0.63	0.010	0.099	0.013	0.50/0.75	0.60/0.65	0.01	1.61
**Task performance (TP)**	0.54	0.010	0.100	0.010	0.41/0.66	0.52 /0.56	0.07	14.89
TP-administrative	0.38	0.003	0.059	0.024	0.31/0.46	0.34/0.43	0	0.00
TP-administrative- multi-item	0.38	0.003	0.059	0.025	0.31/0.45	0.33/0.43	0.04	11.76
TP-research	0.62	0.001	0.032	0.003	0.58/0.66	0.61/0.63	0.10	19.23
TP-research-multi-item	0.65	0.008	0.087	0.010	0.54/0.76	0.63/0.67	0.13	25.00
TP-research-mono-item	0.52	0.017	0.132	0.032	0.35/0.69	0.46/0.58	0.01	1.96
**Contextual performance (CP)**	0.51	0.030	0.174	0.026	0.29/0.73	0.46/0.56	0.08	18.60
CP-administrative	0.36	0.005	0.072	0.030	0.26/0.45	0.30/0.41	0	0.00
CP-research	0.59	0.027	0.165	0.027	0.38/0.80	0.54/0.64	0.03	5.36
CP-research-multi-item	0.61	0.019	0.137	0.029	0.43/0.79	0.55/0.67	0.05	8.93
CP-research-mono-item	0.57	0.035	0.188	0.050	0.33/0.81	0.47/0.67	0.01	1.79
**Positive performance (PP)**	0.49	0.023	0.151	0.048	0.30/0.68	0.40/0.58	0.01	2.08
PP-research	0.48	0.025	0.159	0.056	0.27/0.68	0.37/0.59	0	0.00

*R_YY_, weighted-sample average interrater reliability corrected for direct range restriction; VAR_RYY_, weighted-sample average variance of R_YY_; SD_RYY_, standard deviation of the R_YY_; 80%CrI, 80% credibility interval of R_YY_; 95%CI, 95% confidence interval of R_YY_; Δ, Increment of reliability size due to range restriction; %Δ, Percentage of increment of reliability due to range restriction correction; OJR, overall job performance ratings; TP, task performance ratings; CP, contextual performance ratings; PP, positive performance ratings*.

As can be seen, the average observed interrater reliability of overall job performance ratings was 0.56 for the total cumulated sample size. Sampling error explained 14% of the observed variability, which suggests that the moderator analysis is appropriate. In this analysis, the appraisal purpose was shown to be a moderator of the observed interrater reliability. When the ratings had been done for administrative purposes, the observed interrater reliability fell to 0.45, but the observed interrater reliability rose to 0.61 when the ratings had been done for research purposes.

The corrected interrater reliability estimates were 0.61 for overall job performance, 0.45 for the ratings collected for administrative purposes, and 0.69 for research purposes. The respective 90% credibility values were 0.40, 0.30, and 0.54 and the respective values of the lower bound of the 95% confidence interval were 0.59, 0.40, and 0.67. These estimates mean that the interrater reliability generalizes across samples and that the corrected mean interrater reliability is statistically different from zero with a probability of 97.5%.

As can be see, there are 16 correlations points when comparing the observed interrater estimates of the ratings collected for administrative and research purposes, and 24 correlations points in the corrected estimates for these two subgroups. To test whether the two interrater reliability estimates are statistically different, we calculated the 95% confidence values (lower bound of the 90% confidence interval) of the difference of the observed and corrected estimates. If this value is positive, then the 95% probability that the two estimates are different is supported and we can conclude that the appraisal purpose is a moderator of the interrater reliability. As the pooled weighted SE of the difference is 0.01 in both cases, the 95% confidence values are 0.14 and 0.22, respectively, which supports Hypothesis 1 that the appraisal purpose is a moderator of the interrater reliability of the overall performance ratings. It can also be seen that the 95% confidence intervals do not overlap.

Hypothesis 2 posits that the type of scale (mono-item vs. multi-item) is another moderator of interrater reliability. In order to test this hypothesis it was necessary to conduct a hierarchical meta-analysis which examined the effects of the combination of the two moderators, i.e., purpose and scale type. For overall job performance, we were able to conduct three hierarchical meta-analyses: for the combination of research purpose- mono-item scales, for research purpose-multi-item scales, and for administrative-purpose-multi-item scales. We did not have enough studies for the combination administrative purpose-mono-item scales. Tables 3, 4 report the interrater reliability results for the combination of the moderators. As can be seen, the corrected interrater reliability estimate for the combination of research purpose-multi-item scale is larger than the estimate for the combination of administrative purpose-multi-item scale (difference = 0.25; pooled SE = 0.01), and the corrected estimate for the combination of multi-item scale-and- research purpose ratings is larger than the corrected estimate for the combination of mono-item-and-research purpose ratings (difference = 0.07; pooled SE = 0.01). Next, we calculated the 95% confidence value of these two differences and we found a 95% confidence value of 0.23 for the first difference and a 95% confidence value of 0.05 for the second difference. In addition, the 95% confidence intervals do not overlap. Consequently, we conclude (1) that the number of items of the job performance measure is a moderator of the interrater reliability of overall job performance ratings collected for research purposes, and (2) that there is an interaction between the two moderators (i.e., purposes X scale type). In other words, the number of items appears to be a factor in the agreement between raters. Therefore, Hypothesis 2 was partially supported.

The comparison between the observed and the corrected estimates indicates that range restriction was also a determinant of the interrater reliability of overall performance ratings collected for research purposes. Range restriction proved to be responsible for a 13% shrinkage in the reliability of the ratings collected for research purposes (z difference = 9.06), but it has no effect on the ratings collected for administrative purposes (z difference = 0).

The findings for task performance showed a similar pattern to the findings for overall job performance, with the difference that the reliability values are smaller for the three conditions, i.e., for administrative and research purposes, and the totality of the coefficients. The average observed interrater reliability of task performance ratings was 0.47 for the total cumulated sample size, and sampling error explained 20.8% of the observed variability, which suggests that the moderator analysis is appropriate. The 80% credibility interval is 0.25, which is wide enough to support the analysis of moderators. The observed interrater reliability for the administrative-purpose ratings was 0.38 and it was 0.52 for the research-purpose ratings, and the respective corrected estimates were 0.38 and 0.62, which suggested that the appraisal purpose was also a moderator of the interrater reliability for task performance ratings. The 90% credibility values were 0.41, 0.31, and 0.58 for task performance ratings as a whole and for administrative-purpose and research-purpose ratings, respectively. The lower bound of the 95% confidence intervals was 0.52, 0.34, and 0.61, for task performance, for the administrative ratings and for research ratings. Therefore, the interrater reliability generalizes across samples and is statistically different from zero with a probability of 97.5%.

The 95% confidence values of the difference of the corrected estimates were 0.23 (pooled SE = 0.007), 0.25 (pooled SE = 0.01), and 0.10 (pooled SE = 0.017) for the comparisons between research and administrative ratings, research-multi-item vs. administrative- multi-item, and research-multi-item vs. research-mono-item ratings. The 95% confidence intervals do not overlap. As in the case of overall job performance, the results for task performance showed that the number of items of the measure was a moderator of the interrater reliability of overall job performance ratings. Therefore, Hypothesis 1 was fully supported and Hypothesis 2 received partial support.

The comparison between the observed and the corrected estimates indicates that range restriction attenuated the interrater reliability of the task performance ratings collected for research purposes. Range restriction was shown to be responsible for a 19% shrinkage in the reliability of the ratings collected for research purposes (z difference = 6.4), but it didn't have any effect on the ratings collected for administrative purposes (z difference = 0).

Concerning contextual performance, the observed interrater reliability was 0.43 for the whole set of coefficients and sampling error explained slightly less than 9% of the observed variability. Therefore, the moderator analysis seems to be appropriate. Beginning with the purpose of ratings as a moderator, the observed interrater reliability for the administrate ratings was 0.36 vs. 0.56 for the research purpose ratings. The respective corrected values were 0.51 for contextual performance, and 0.36 and 0.59 for administrative vs. research purposes ratings. The 90% credibility values were 0.28, 0.26, and 0.38 for contextual performance ratings as a whole and for administrative-purpose and research-purpose ratings, respectively. Therefore, the interrater reliability generalizes across samples. The width of the credibility interval was particularly large for task performance (44 correlation points) and the 90% credibility interval for administrative ratings and research ratings were large, too. The lower bounds of the 95% confidence intervals were 0.46, 0.30, and 0.54, for task performance, for the administrative ratings and research ratings, respectively. Therefore, the interrater reliability generalizes across samples and is statistically different from zero with a probability of 97.5%.

Taking into account that the difference between the interrater estimates for administrative vs. research ratings of contextual performance was large (0.36 vs. 0.59), we calculated the 95% confidence value of the difference of the corrected estimates. The pooled SE was 0.027 and the 95% confidence value was 0.19, which supports Hypothesis 1 that the purpose of the ratings is a moderator of the interrater reliability of contextual performance. As for the second moderator, the type of scale, we were able to analyze the difference in the case of the ratings collected for research purposes only. The 95% confidence value was −0.02, which indicated that the number of items of the scale did not moderate the interrater reliability for the research ratings. Therefore, Hypothesis 2 was not supported in this case.

The comparison between the observed and the corrected interrater reliability of the contextual performance ratings showed that range restriction reduced the interrater reliability by about 19%, but the 95% confidence interval of the difference of means included zero in the case of both administrative ratings and research ratings, which suggests that range restriction did not affect the interrater reliability when the analyses are done with the ratings purpose as a moderator.

Concerning the interrater reliability of positive performance ratings, Tables 4, 5 report only the results of the total set of coefficients and for the ratings collected for research purposes, as we found only two studies of ratings collected for administrative purposes. In both cases the observed estimates of the interrater reliability were 0.48, due to the fact that the ratings collected for research purposes represented 92% of the total sample for the positive performance category. Sampling error explained 11.17% of the observed variability, but the moderator analysis cannot be done in this category as we do not have enough estimates. The 90% CVs were 0.30 and 0.27, respectively and both the 80% credibility interval and the 95% confidence interval did not include zero. Therefore, the generalization of the interrater reliability was supported and the interrater mean is different from zero. Range restriction was not a relevant factor in the estimates of the interrater reliability for this performance dimension.

**Table 5:** Comparisons among the average interrater reliability estimates for the four job performance dimensions.

**Comparison**	**Interrater reliability difference**	**SE_**diff**_**	**95% confidence value**
OJP–TP	0.07	0.0107	0.052
OJP–CP	0.10	0.0146	0.076
OJP–PP	0.12	0.0147	0.086
TP–CP	0.03	0.0168	0.002
TP–PP	0.05	0.0177	0.020
CP–PP	0.02	0.0310	−0.030

*OJP, overall job performance; TP, task performance; CP, contextual performance; PP, positive performance. SE_dif_, standard error of the difference of interrater reliability estimates; 95% Conf Value, confidence value of 95% (lower bound of the 90% confidence interval)*.

The last analyses we carried out were the comparisons among the average interrater reliability estimates for the four job performance dimensions in order to answer the question of whether some dimensions were more reliably rated than others. Consequently, we calculated the 95% confidence value of the difference between pairs of interrater reliability estimates. In other words, we compared the interrater reliability of overall job performance with the interrater reliability of task, contextual, and positive performance dimensions; we compared the interrater reliability of task performance with the interrater reliability of contextual and positive performance, and, finally, we compared the interrater reliability of contextual and positive performance. Table 5 reports the results of these comparisons. The 95% confidence values of the difference among interrater reliability estimates showed overall job performance was more reliably rated than the other three dimensions, followed by task performance. The 95% confidence values were positive in five cases out six. We did not find statistical differences between contextual and positive performance. Therefore, these findings supported the suggestion that some dimensions are more difficult to rate than others (Borman, 1979; Wohlers and London, 1989; Viswesvaran et al., 1996). From this point of view, our findings suggest that overall job performance is easier to evaluate than the other three dimensions and that the most reliable method is to evaluate overall job performance for research purpose ratings with a multi-item scale (corrected interrater reliability = 0.70).

In summary, as a whole, the results of this interrater-reliability meta-analysis showed that the rating purpose is a moderator of the interrater reliability of overall job performance, task performance, and contextual performance, which confirmed Hypothesis 1. The difference size of the corrected interrater reliability for administrative ratings and research ratings was 0.24 in the three job performance dimensions, which in percentage is a difference of 53, 63, and 64% for overall, task, and contextual performance, respectively. The best estimates of observed interrater reliability were 0.56, 0.47, 0.43, and 0.48 for overall, task, contextual and positive performance dimensions. The respective best corrected estimates for the same performance dimensions were 0.61, 0.54, 0.51, and 0.49. For the ratings collected for administrative purposes, the best observed estimates of interrater reliability were 0.45, 0.38, and 0.36 for overall, task, and contextual performance. The best observed estimates of the ratings collected for research purposes were 0.61, 0.52, 0.56, and 0.48 for overall, task, contextual, and positive performance, and 0.69, 0.62, 0.59, and 0.48 were the best corrected estimates of the interrater reliability of overall, task, contextual, and positive performance ratings. In second place, we found that the hypothesis that the scale type might be a moderator of the interrater reliability of job performance ratings received partial support as multi-item scales showed larger interrater reliability for overall job performance and task performance, but we did not find any difference between multi vs. mono-item scales for contextual performance ratings. In third place, range restriction was an artifactual factor that reduced the true interrater reliability of overall, task, and contextual performance ratings. Finally, we found that overall job performance is easier to evaluate than the other three dimensions as it has the largest interrater reliability estimate.

### Publication Bias Analysis

As mentioned in section Method for Reliability Generalization of Interrater Coefficients of Job Performance, we used four methods to detect potential publication bias. For the separate meta-analyses of the published and unpublished studies, we used the larger dataset of research ratings of overall job performance, which includes 201 reliability coefficients. We found that the average corrected mean and the SD were the exactly same for published and unpublished studies (average corrected *r*_*yy*_ = 0.69; *SD* = 0.14). Therefore, at least for this subgroup of coefficients, we can discard the idea that the source of publication distorts the interrater reliability estimate. We cannot conduct separate analyses for the administrative ratings because the 18 coefficients were obtained from published studies.

The second analysis was to obtain the Pearson correlation between the year of the study and the interrater estimate size. We carried out this analysis with the full data set of 219 independent coefficients of overall job performance. The correlation was −0.08 (*p* = 0.26). Therefore, there is no relationship between the year of the study and the reliability size. The correlation between the study year and the sample size was 0.09 (*p* = 0.163), which indicates that the size of the samples in the reliability studies was relatively similar for the years covered in this meta-analysis.

In third place, as requested by a reviewer, we also calculated the failsafe number (N), where N would be the number of studies not included in the meta-analysis with a reliability of zero which reduce the average reliability to a specific estimate, e.g., a half or a third of the obtained estimate. Orwin, (1983; but see also Schmidt and Hunter, 2015, p. 533–534) derived the formula of N, and Becker (2005) and Schmidt and Hunter (2015) discussed some limitations of the failsafe N. Table 6 reports the estimates for three hypothetical reduced estimates of the failsafe N. The failsafe N showed that the number of required studies with reliability of zero (“lost” studies) would be highly unlikely.

**Table 6:** Results of Orwin's test of failsafe N.

**Study**	**K**	**R_**YY**_**	**R.75**	**R.5**	**R.25**	**N75**	**N50**	**N25**
OJP	219	0.60	0.150	0.30	0.450	657	219	73
OJP-administrative	18	0.42	0.105	0.21	0.315	54	18	6
OJP-administrative-multi-item	14	0.41	0.102	0.20	0.307	42	14	5
OJP-research	201	0.69	0.172	0.34	0.517	603	201	67
OJP-research-multi-item	145	0.70	0.175	0.35	0.525	435	145	43
OJP-research-mono-item	56	0.63	0.155	0.31	0.472	168	56	19
TP	94	0.52	0.130	0.26	0.390	282	94	31
TP-administrative	6	0.35	0.085	0.17	0.262	18	6	2
TP-administrative-multi-item	5	0.35	0.087	0.17	0.262	15	5	2
TP-Research	88	0.62	0.155	0.31	0.465	264	88	29
TP-research-multi-item	71	0.65	0.165	0.32	0.487	213	71	24
TP-research-mono-item	17	0.52	0.130	0.26	0.390	51	17	6
CP	43	0.51	0.125	0.25	0.382	129	43	14
CP-administrative	6	0.36	0.090	0.18	0.270	18	6	2
CP-research	37	0.59	0.145	0.29	0.442	111	37	12
CP-research-multi-item	23	0.61	0.152	0.30	0.457	69	23	8
CP-research-mono-item	14	0.57	0.142	0.28	0.427	42	14	5
PP	10	0.49	0.122	0.24	0.367	30	10	3
PP-research	8	0.48	0.120	0.24	0.360	24	8	3

*OJP, overall job performance; Administrative, administrative-purpose ratings; Research, research-purpose ratings; TP, task performance; CP, contextual performance; PP, positive performance; K, number of original studies; R_YY_, interrater reliability; R-75, hypothetical value of interrater reliability if the original one is attenuated 75%; R.50, hypothetical value of the interrater if the original one is attenuated 50%; R.25, hypothetical value of interrater reliability if the original one is attenuated 25%; N75, number of studies with interrater reliability of zero that would have to be added to the dataset to obtain the hypothetical interrater reliability of R.75; N50, number of studies with interrater reliability of zero that would have to be added to the dataset to obtain the hypothetical interrater reliability of R.50; N25, number of studies with interrater reliability of zero that would have to be added to the dataset to obtain the hypothetical interrater reliability of R.25*.

Our fourth strategy for analyzing publication bias was cumulative meta-analysis. Borenstein (2005), Kepes et al. (2012), and Schmidt and Hunter (2015) posited that the cumulative meta-analysis is the most powerful method for detecting and estimating publication bias. Cumulative meta-analysis consists of a series of meta-analyses in which new studies are added one-by-one and a new average estimate is calculated with the addition of each new study. Both Borenstein (2005) and Schmidt and Hunter (2015) suggest that the studies should be ranked from the study with the largest sample size to the study with the smallest sample size. In the present case, the interrater reliability estimate and its standard error are calculated and a moving forest plot is developed. The absence of publication bias is observed when a sustained line is described after some studies are added. On the contrary, if the addition of small-sample studies changed the average estimate and its standard error, this would be evidence of publication bias. Cumulative forest plots were developed for the main meta-analyses.

Supplementary Figures 1–6 present the results of the cumulative meta-analyses carried out to examine the potential publication bias. As can be seen in the figures, the cumulative meta-analyses conducted with the corrected interrater reliability estimates showed evidence against publication bias for all the job performance categories and the research purpose. The point estimate established very rapidly in all the cases, and it did not shift with the inclusion of additional studies.

Therefore, the results of the four approaches concur that publication bias is not a relevant issue in the current study.

## Discussion

Interrater reliability of supervisory performance ratings has recently been the focus of considerable debate (LeBreton et al., 2003; Murphy, 2008, 2014; Burke et al., 2014; Sackett, 2014; Viswesvaran et al., 2014; Salgado et al., 2016). The interrater reliability estimates of supervisory performance ratings have been criticized in connection with their use to correct the validity coefficients of the assessment procedures (e.g., cognitive tests, personality inventories) used for predicting job performance in personnel selection. Some researchers have pointed out that the correction for low interrater reliability produces inflated validity coefficients (LeBreton et al., 2003, 2014) and other researchers have suggested that interrater coefficients should not be used because they might be estimates of validity rather than of reliability (Murphy and De Shon, 2000; Murphy, 2008).

Two hypotheses were examined in connection with two potential moderating variables of the interrater reliability of job performance ratings. The first moderator was the appraisal purpose. It was hypothesized that supervisors would show larger interrater reliability when their ratings were made for research purposes (e.g., to be used as a criterion in validation studies) because they would not be affected by personal and situational influences (or the effects of these variables would be relatively small). On the contrary, the personal, contextual, and situational influences would significantly affect the supervisory ratings when they were made for administrative purposes (e.g., promotions, compensation, and feedback). The second moderator analyzed was the type of rating measures of performance, i.e., mono vs. multi-item scales. For this moderating variable, the hypothesis was that the multi-item scales would show larger reliability estimates than the mono-item scales. In addition, we examined whether range restriction produced homogeneity in the ratings and, consequently, the interrater reliability coefficients would appear as lower than they are in reality. Finally, we compared the interrater reliabilities of four performance dimensions to examine if some of the dimensions were easier to evaluate than others and, therefore, the interrater reliability might be different.

The first and most important conclusion of this reliability generalization effort is that, without a doubt, supervisory job performance ratings are reliable and useful when they are collected for research purposes (e.g., for validation studies). However, the supervisory performance ratings can have a more limited reliability and utility for administrative purposes, if several important points discussed below are not taken into account.

The second conclusion is that the appraisal purpose (i.e., administrative vs. research) has a significant effect on the interrater reliability for at least overall, task, and contextual performance dimensions. Concerning the influence of the appraisal purpose, the corrected interrater reliability of overall job performance is 53% larger for research ratings compared with administrative ratings (36% larger in the case of observed interrater reliability). A similar pattern of findings was found for the ratings of task performance and contextual performance. Both the 80% credibility intervals and the 95% confidence intervals supported the hypothesis that the appraisal purpose was a moderator of the interrater reliability. Thus, an important conclusion of this study is that the appraisal purpose is a determinant of the interrater reliability of supervisory performance ratings. Consequently, one important recommendation would be to avoid using interrater reliability estimates without previously considering their evaluation purpose.

The second moderator examined was the type of measure, i.e., mono-item vs. multi-item scales. To examine the specific effects of this potential moderator we conducted a series of hierarchical sub-grouping meta-analyses as recommended by Schmidt and Hunter (2015), in order to avoid potential errors of interpretation about the respective effects of the two moderators on the interrater reliability. If the two moderators have interacting effects, only the hierarchical analysis provides a correct basis for conclusions. We found that the scale type is a moderator of the interrater reliability in the case of overall job performance ratings and task performance ratings collected for research purposes. As shown by the 95% confidence value of the difference between the estimates, the corrected interrater reliability is larger for the multi-item scales than for the mono-item scales. Therefore, researchers should be aware that there are gains to be made in interrater reliability by using multi-item scales instead of mono-item scales. As no effects were found for contextual performance, the last conclusion applies only to the interrater reliability of overall job performance and task performance, and it is not applicable to other dimensions and facets of performance (e.g., contextual performance and positive performance).

In our view, multi-item scales should be preferred to mono-item measures, because they capture more construct variance of the performance space. Nevertheless, mono-item measures of overall job performance can be useful for validation purposes as they are reliable if the purpose is research.

Concerning the restriction of range, this artifact has some effect on the size of the interrater reliability estimates when the ratings are collected for research purposes, but we did not find effects on the ratings collected for administrative purposes. Thus, the low interrater reliability estimates found typically in research studies can also be a partial consequence of this statistical artifact. On average, range restriction is responsible for 13% of the reduction in the size of interrater reliability of supervisory ratings of overall job performance collected for research purposes using multi-item scales. Similar results were found for task performance, and, to a lesser extent, for contextual performance ratings. Range restriction has practically no effect on the combination of ratings collected for research purposes using mono-item scales (about 2% of reduction). Therefore, the results of this reliability generalization study partially support the hypothesis that low interrater reliability is partially due to the variance homogeneity produced by range restriction (LeBreton et al., 2003; Murphy, 2008; Viswesvaran et al., 2014), but only in the case of research ratings obtained with multi-item scales. Furthermore, this effect on the interrater reliability is independent of the effect of the appraisal purpose.

Although we did not state any hypothesis in advance concerning the idea that supervisors have less difficulty reaching agreement on some performance dimensions than others (Borman, 1979; Wohlers and London, 1989; Viswesvaran et al., 1996; Sáez, 2007), some findings of the present study suggest that this is the case at least for overall job performance and task performance. Comparatively, overall job performance ratings are more reliably rated than the other three dimensions. This is an additional contribution of this meta-analytic study. This finding can be partially related to the bandwidth-fidelity debate in psychological assessment (Cronbach, 1990; Salgado, 2017). Some studies in personality and cognitive assessment have shown that bandwidth instruments have larger reliability and larger criterion-oriented validity than narrower measures (Ree et al., 1994; Salgado et al., 2013, 2015b; Salgado, 2017; Harari et al., 2019). This seems to be the case in the dimensions examined in this reliability generalization study, as the narrower dimensions (task, contextual, and positive performance) showed smaller interrater reliability than overall job performance. The finding that overall job performance has larger interrater reliability contradicts the previous finding of Viswesvaran et al. (1996) that narrower measures of performance (e.g., productivity, quality, administrative competence, and compliance with and acceptance of authority) have larger interrater reliability than overall job performance.

Two additional findings must be mentioned. The first one is about publication bias and the second has to do with the unpublished estimates of interrater reliability obtained from the technical reports of the General Aptitude Test Battery (GATB). Concerning the first point, we do not find evidence of publication bias in our data set. We used four methods to analyze potential publication bias and the four methods concurred that publication bias seems to be absent in our database. Moreover, we found that the publication year of the study did not correlate with the reliability size and that the magnitude of the interrater reliability was very similar across seven decades when we controlled for the effects of moderators.

It should also be noted that the technical reports of the GATB provided 45 unpublished interrater reliability estimates and that these coefficients were not used previously in a reliability generalization study. For instance, they were not used in the meta-analysis of Viswesvaran et al. (1996). As a whole, this set of interrater reliability coefficients showed an average reliability of 0.70, which is considerably larger than the interrater reliability of 0.52 for published studies found by Viswesvaran et al. (1996). Two possible explanations for the divergence are: (1) that all the studies of the GATB used ratings collected for research purposes, which is not the case of the whole dataset of Viswesvaran et al. (1996), which also included studies with ratings collected for administrative purposes; (2) that the conditions for collecting the ratings across the organizations were relatively homogeneous, as the technicians of the U.S. Personnel Office trained and helped the organizations and managers during the processes of performance appraisal, which contrasts with the heterogeneity of the conditions and rater training in the studies included in Viswesvaran et al.'s (1996) database. Moreover, it must be noted that the GATB set of studies was an important contribution toward establishing that the average reliability for published and unpublished studies was the same.

### Implications for Research and Practice

The current findings have relevant consequences for local validation studies and for meta-analyses when researchers and practitioners have no estimates of the interrater reliability of the job performance ratings. Some previous meta-analyses developed specific empirical distributions of interrater reliability of job performance ratings based on the coefficients of the primary studies included in those meta-analyses (e.g., Salgado et al., 2003), whereas other meta-analyses have assumed the interrater reliability (e.g., Hunter and Hunter, 1984; Barrick and Mount, 1991). Also, primary studies used an assumed reliability coefficient (e.g., Rodríguez and López-Basterra, 2018). The results found in the present study can serve to clarify what interrater reliability estimate should be used to correct validity for criterion unreliability. In order to apply the range restriction correction, the researcher should identify firstly if the study used performance measures collected for research purposes or if they have been collected for administrative purposes. In second place, the researcher and the practitioner should identify if the study was conducted with mono-item or multi-item scales. Next, the researcher should use the appropriate estimate.

In selecting the appropriate estimate, it is necessary to clarify an important distinction. Corrected interrater reliability is the job performance reliability in the population of applicants and observed interrater reliability is the reliability of job performance in the population of incumbents (i.e., employees).

Researchers should be aware that observed interrater reliability is of interest when an appraisal instrument is only applied and used in a restricted population (i.e., incumbents). For example, if a company develops a performance assessment for employees, this company, for this purpose, will not be interested in the interrater reliability of the instrument in the population of applicants containing both employees and rejected candidates (Fife et al., 2012). Similarly, if the organization develops a measure of contextual performance to be used with employees, it would be interested in the interrater reliability of the instrument in the population of applicants.On the contrary, if the organization is interested in applicant population job performance, then the corrected interrater reliability would be of interest.

With regard to the last point, it is important to stress that if the range restriction is indirect (which is the most frequent case in personnel selection), the observed interrater reliability must be used to correct the observed validity coefficients. Therefore, for instance, if the criterion was overall job performance, then 0.61 should be used for the ratings collected for research purposes and 0.45 for the ratings collected for administrative purposes. However, as Fife et al. (2012) pointed out, when multiple- hurdle procedures are used (a frequent case in personnel selection) or when the range restriction is direct (a less frequent case), then the corrected reliability value should be used. In these cases, 0.70 should be used when the criterion was collected for research purposes and 0.45 when it was collected for administrative purposes if the criterion was overall job performance. Finally, these figures can be used to create the appropriate distributions of criterion interrater reliability.

In future research on the reliability of supervisory performance ratings, it will be essential to distinguish between ratings as a dependent variable (e.g., criterion) and ratings as an independent variable (e.g., predictor). The respective interrater reliability coefficients are clearly different. The present study has demonstrated that supervisory performance ratings can be a reliable instrument for research purposes, particularly when they are used as a criterion (e.g., in validation studies). For administrative purposes (e.g., decisions on promotion, compensation, and so on), three or more supervisors would be required to obtain an acceptably reliable measure of job performance.

### Limitations

Like other meta-analytic studies, the present one has some limitations that must be mentioned. One limitation of this interrater reliability generalization study is that some categories have a small number of studies. Consequently, we were not able to perform some hierarchical meta-analyses. Future research should address this concern and reduce the second-order sampling error present in these analyses. A second limitation is that, although we have made a significant effort to obtain studies from different countries and languages, the majority of the studies were conducted in the US and English was the most frequently used language. It would be desirable to extend the number of countries and languages in future meta-analyses.

A third limitation is that although we have tried to examine the potential role of the type of scales (mono vs. multi-item), we were not able to perform this analysis in a number of cases due to the absence of studies. In addition, it would be worthwhile to analyze whether different multi-item scales, such as graphic scales, behaviorally-anchored rating scales, behavior checklist scales, and so forth (see Aguinis, 2013) produce similar results.

A fourth limitation is that we were not able to examine if occupation type (e.g., police; manager) or some occupational characteristics (e.g., job complexity) moderate interrater reliability. For instance Hirsh et al. (1986) speculated that the low validity of general mental ability tests for predicting job performance in law enforcement occupations may be due to the difficulties raters have in observing behavior in these occupations. Concerning job complexity, as the level of information-processing increases, more potential solutions (and behaviors) may be possible for the same issue, and consequently, this might make agreement among raters more difficult. In order to examine the moderating effects of job complexity, we would need many additional studies to conduct a fully hierarchical meta-analysis.

## Conclusion

In summary, the present reliability generalization study has shown that the interrater reliability of supervisory performance ratings is affected by two moderators, the purpose of the ratings and the type of scales used in the assessment process. Moreover, we found range restriction attenuated the interrater reliability of research-collected ratings and that overall job performance showed higher interrater reliability than the other three performance dimensions. We found that the best estimate of the observed interrater reliability of supervisor ratings of overall job performance is 0.61 if the ratings are collected for research purposes and 0.45 if they are for administrative purposes. The respective corrected estimates are 0.70 for research purposes and 0.45 for administrative purposes. The study also showed that the best estimates of the corrected interrater reliability for task performance, contextual performance, and positive work behavior are 0.62, 0.59, and 0.48, respectively, when they are collected for research purposes, and 0.30 for task performance and contextual performance, when the ratings are for administrative purposes. Moreover, the meta-analysis showed that multi-item scales should be preferred to mono-item scales, as the corrected interrater reliability of the former is larger. Finally, we argued that observed interrater reliability should be used in local validation studies and meta-analyses when the range restriction of job performance is indirect and that when the range restriction is direct and when a multiple hurdle process has been used, then the corrected interrater estimates should be used.

## Data Availability Statement

All datasets generated for this study are included in the manuscript/Supplementary Files.

## Author Contributions

Both authors listed have made a substantial, direct and intellectual contribution to the work, and approved it for publication.

### Conflict of Interest

The authors declare that the research was conducted in the absence of any commercial or financial relationships that could be construed as a potential conflict of interest.
