# Supplementary material for: Meta-Analysis of Interrater Reliability of Supervisory Performance Ratings: Effects of Appraisal Purpose, Scale Type, and Range Restriction
Source: Front Psychol. 2019 Oct 18;10:2281. doi: 10.3389/fpsyg.2019.02281 (PMC6813221; doi:10.3389/fpsyg.2019.02281)
Supplement: Supplementary file 1 [file Data_Sheet_1.PDF]

Appendix 1. Interrater Reliability Coefficients of Supervisory Ratings of Overall Performanc.

Study	N	r_{yy}	Type	Purpose	*u*
Albrecht et al. (1964)	31	.63	2	1	1
Alessandri & Vecchione (2012)	201	.65	2	2	1
Altink (1999)	64	.65	2	1	1
Bass & Turner (1973)	368	.62	2	2	1
Bass et al. (2003)	72	.75	1	1	1
Bass & Wurster (1953)	123	.49	1	2	1
Bernardin et al. (1980)	94	.64	2	1	1
Bickle et al. (2008)	10	.63	2	1	1
Blicke et al. (2011)	79	.58	2	1	1
Blumenfeld & Janus (1974)	65	.68	1	1	1
Bonnardel (1949)	36	.60	1	1	1
Bonnardel (1949)	43	.75	1	1	1
Borman et al. (1975)	493	.48	2	1	1
Borman et al. (1976)	14	.50	2	1	1
Burroughs (1996)	38	.89	2	1	1
Bynum et al. (2013)	5278	.33	2	2	1
Campbell (1986) Study 1	141	.66	2	1	1
Campbell (1986) Study 2	154	.61	2	1	1
Campbell (1986) Study 3	113	.63	2	1	1
Campbell (1986) Study 4	149	.66	2	1	1
Campbell (1986) Study 5	154	.80	2	1	1
Campbell (1986) Study 6	126	.66	2	1	1
Campbell (1986) Study 7	143	.76	2	1	1
Campbell (1986) Study 8	156	.78	2	1	1
Campion et al. (1988)	30	.76	2	1	.65
Campion et al. (1994)	70	.65	2	2	1
Cascio & Valenzi (1978)	952	.74	2	2	1
Cellar et al. (1996)	424	.74	2	1	1
Cocanougher & Ivancevich (1978)	91	.60	2	1	1
Cooper & Payne (1967)	135	.61	1	1	1
Cooper (1966)	57	.83	2	1	1
Crook et al. (2011) Study 1	44	.73	2	1	1
Crook et al. (2011) Study 2	113	.72	2	1	1
Cummins (1971)	133	.44	1	1	1
Day & Silverman (1989)	43	.59	2	1	1
Denton (1963)	96	.84	1	1	.71
Dicken & Black (1965)	57	.56	1	1	1

Distefano & Bass (1959) Study 1	10	.87	2	1	1
DistefanoBass (1959) Study 2	7	.40	2	1	1
Dunnete & Kirchner (1960)	6	.68	1	1	1
Dunnette & Motowidlo (1976) Study 1	700	.53	1	1	1
Dunnette & Motowidlo (1976) Study 2	415	.54	1	1	1
Dunnette & Motowidlo (1976) Study 3	362	.57	1	1	1
Dunnette & Motowidlo (1976) Study 4	204	.55	1	1	1
Fineman & Payne (1974)	6	.83	1	1	1
Finley et al. (1977)	173	.47	2	1	1
Friedland & Michael (1987)	147	.37	2	1	1
Goguelin (1953)	67	.74	1	1	1
Gough et al. (1991) Study 2	20	.47	1	1	1
Gough et al. (1991) Study 1	95	.64	1	1	1
Guilford & Comrey (1948)	242	.66	2	2	1
Guion (1965)	48	.76	1	1	1
Gunderson & Nelson (1966)	139	.46	2	1	1
Gunderson & Ryman (1971)	197	.51	2	1	1
Hackman & Porter (1968)	82	.60	2	1	1
Handyside & Duncan (1954)	34	.60	1	1	.75
Harris et al. (1995)	55	.67	2	1	1
Hilton et al. (1955)	72	.47	1	1	1
Hogan et al. (1984)	100	.60	2	1	1
Hogan et al. (1992)	74	.57	2	1	.82
Hough (1984)	153	.64	2	1	1
Hueber (1954)	27	.55	2	1	1
Hughes & Prien (1986)	49	.42	2	1	1
Ivancevich (1977)	102	.54	2	1	1
Jackofsky et al. (1986)	139	.62	1	2	.80
Judge & Erez (2007)	122	.51	2	1	1
Kaiser & Craig (2005)	1404	.26	2	2	1
Kaufman (1972)	73	.61	2	2	1
Keller (2006)	118	.50	1	1	1
Knauft (1949)	35	.68	2	1	.58
Lado & Alonso (2017)	61	.80	2	1	1
Landy et al. (1976)	342	.49	2	1	1
Lawshe & Ginley (1951)	18	.82	1	1	1
Lee et al. (1981)	144	.52	2	1	1
Levine & Baker (1992)	373	.71	2	1	1
Levy & Stene (1965)	11	.83	1	1	1
Lewis (1960)	48	.50	1	1	1
Lowmaster & Morey (2012)	59	.50	2	2	.70
Lowry (1994)	55	.45	1	1	1
Mandell & Adkins (1946)	63	.65	2	1	1

Maxim & Dielman (1987)	303	.32	2	2	1
Miner (1970)	25	.75	1	2	1
Mitchel & Albright (1972)	66	.65	1	1	1
Mount et al. (1997)	2297	.45	2	2	1
Nealy & Owen (1970)	25	.75	2	1	1
Pavett & Lau (1982)	48	.67	1	2	1
Petrie & Powell (1951)	126	.65	2	1	1
Prien & Liske (1962)	116	.60	2	1	1
Pynes & Bernardin (1989)	190	.56	2	1	.70
Robertson et al. (1993)	89	.63	2	1	1
Rothstein (1990) Sample 1	51	.36	2	1	.53
Rothstein (1990) Sample 2	76	.46	2	1	.67
Rothstein (1990) Sample 3	73	.45	2	1	.67
Rothstein (1990) Sample 4	68	.52	2	1	.54
Rothstein (1990) Sample 5	102	.45	2	1	.59
Rothstein (1990) Sample 6	105	.60	2	1	.69
Rothstein (1990) Sample 7	104	.48	2	1	.66
Rothstein (1990) Sample 8	130	.55	2	1	.62
Rothstein (1990) Sample 9	106	.62	2	1	.56
Rothstein (1990) Sample 10	97	.44	2	1	.55
Rothstein (1990) Sample 11	111	.56	2	1	.66
Rothstein (1990) Sample 12	134	.60	2	1	.69
Rothstein (1990) Sample 13	315	.56	2	1	.66
Rothstein (1990) Sample 14	322	.60	2	1	.70
Rothstein (1990) Sample 15	361	.55	2	1	.67
Rothstein (1990) Sample 16	285	.69	2	1	.69
Rothstein (1990) Sample 17	262	.60	2	1	.71
Rothstein (1990) Sample 18	297	.55	2	1	.68
Rothstein (1990) Sample 19	255	.54	2	1	.68
Rothstein (1990) Sample 20	357	.66	2	1	.74
Rothstein (1990) Sample 21	832	.66	2	1	.77
Rothstein (1990) Sample 22	684	.66	2	1	.77
Rothstein (1990) Sample 23	552	.58	2	1	.72
Rothstein (1990) Sample 24	570	.64	2	1	.75
Rothstein (1990) Sample 25	478	.69	2	1	.79
Rothstein (1990) Sample 26	504	.63	2	1	.78
Rothstein (1990) Sample 27	437	.70	2	1	.76
Rothstein (1990) Sample 28	396	.67	2	1	.78
Rothstein (1990) Sample 29	341	.63	2	1	.79
Rothstein (1990) Sample 30	320	.59	2	1	.75
Rothstein (1990) Sample 31	310	.62	2	1	.76
Rothstein (1990) Sample 32	281	.70	2	1	.77
Rothstein (1990) Sample 33	213	.67	2	1	.78

Rothstein (1990) Sample 34	134	.59	2	1	.75
Rothstein (1990) Sample 35	105	.68	2	1	.70
Rothstein (1990) Sample 36	116	.47	2	1	.73
Rothstein (1990) Sample 37	91	.65	2	1	.90
Russell & Down (1995)	120	.82	2	1	1
Rush (1953)	100	.52	1	1	.50
Sáez (2007)	59	.87	2	1	.88
Salgado & Blanco (1988)	30	.58	2	1	.65
Salgado & Blanco (1990)	8	.73	2	1	1
Salgado (2015)	176	.59	1	2	1
Salgado et al. (2015)	213	.60	2	1	.67
Salgado et al. (2007)	20	.81	2	1	1
Sartain (1946), Sample 1	43	.64	2	1	1
Sartain (1946) sample 2	54	.63	2	1	1
Schippman & Prien (1986)	47	.51	2	1	1
Schuerger et al. (1982)	28	.39	2	2	.77
Scullen et al. (2000)	2142	.45	2	2	1
SHL (1989) Study 22	90	.65	2	1	1
SHL (1989) Study 27	151	.58	2	1	1
SHL (1989) Study 31	60	.72	1	1	1
SHL (1989) Study 47	306	.41	2	1	.76
SHL (1989) Study 52	170	.78	2	1	1
SHL (1989) Study 53	64	.47	2	1	1
SHL (1989) Study 54	91	.49	2	1	1
SHL (1989) Study 68	40	.52	2	1	.66
SHL (1989) Study 69	89	.52	2	1	.60
Siegel (1982)	20	.65	1	2	1
Soar (1956)	29	.88	1	1	1
Spengler (1971)	69	.42	1	1	1
Springer (1953)	100	.66	2	2	1
Sproule & Berkley (2001)	450	.45	2	1	1
Stevens & Campion (1999) Study 1	70	.69	2	1	1
Stevens & Campion (1999) Study 2	72	.68	2	1	1
Taylor (1957)	103	.53	2	1	1
Tenopyr (1969)	126	.57	1	1	1
Thompson & Thompson (1985)	63	.61	1	1	1
Thompson (1970)	71	.35	2	1	1
Tziner & Dolan (1982)	113	.75	1	1	1
Tziner (1984)	188	.75	2	1	.60
Tziner (1984)	67	.82	1	1	1
USES (1951)	50	.79	1	1	1
USES (1954a)	46	.85	1	1	1
USES (1954b)	49	.90	1	1	1

USES (1955)	35	.64	1	1	1
USES (1956a)	57	.62	1	1	1
USES (1956b)	54	.66	1	1	1
USES (1957a)	57	.83	2	1	1
USES (1957b)	63	.62	1	1	1
USES (1957c) Sample 1	39	.57	1	1	1
USES (1957c) Sample 2	38	.79	1	1	1
USES (1957d)	36	.76	1	1	1
USES (1958a)	54	.61	1	1	1
USES (1958b)	51	.84	2	1	1
USES (1958c)	51	.61	2	1	1
USES (1958d)	57	.66	1	1	1
USES (1959a)	113	.82	2	1	1
USES (1959b)	77	.43	2	1	1
USES (1960)	50	.85	1	1	1
USES (1961)	66	.82	2	1	1
USES (1962a)	87	.53	2	1	1
USES (1962b)	110	.48	2	1	1
USES (1962c)	52	.97	2	1	1
USES (1963a)	152	.72	1	1	1
USES (1963b)	25	.67	1	1	1
USES (1963c)	53	.78	1	1	1
USES (1963e)	50	.72	2	1	1
USES (1964)	53	.70	2	1	1
USES (1965a)	84	.89	2	1	1
USES (1965b) Sample 1	34	.75	1	1	1
USES (1965b) Sample 2	27	.72	1	1	1
USES (1965c)	53	.70	2	1	1
USES (1965d)	27	.80	1	1	1
USES (1966)	292	.62	1	1	1
USES (1967) Sample 1	65	.55	2	1	1
USES (1967) Sample 2	83	.78	2	1	1
USES (1968)	50	.82	2	1	1
USES (1969)	50	.56	2	1	1
USES (1970a)	164	.76	2	1	1
USES (1970b)	50	.65	2	1	1
USES (1970c)	51	.77	2	1	1
USES (1970d)	112	.67	2	1	1
USES (1972)	50	.72	2	1	1
USES (1982a)	154	.72	2	1	1
USES (1982b)	855	.56	2	1	1
USES (1982c)	201	.80	2	1	1
USES (19xx) Sample 1	166	.66	2	1	1

USES (19xx) Sample 2	64	.50	2	1	1
Van Iddekinge et al. (2006)	363	.43	2	1	1
Van Scotter & Modowidlo (1994)	141	.66	2	1	.55
Van Scotter & Steel (2000) Study 1	254	.42	2	1	1
Van Scotter & Steel (2000) Study 2	303	.56	2	1	1
Villanova & Bernardin (1990)	56	.77	1	1	1
Wiley (1976)	244	.58	2	1	1
Worbois (1975)	46	.80	2	1	1
Wright & Taw (1999)	10	.57	2	1	1
Zedeck & Baker (1972)	71	.39	2	1	1

Note. N= sample size; r_{yy}= observed interrater reliability coefficient; Type= mono-item (1), multi-item (2); Purpose= research (1), administrative (2); *u*= range restriction value.

Appendix 2. Interrater Reliability Coefficients of Supervisory Ratings of Task Performance.

Study	N	r_{yy}	Type	Purpose	u
Albrecht et al. (1964)	31	.64	2	1	1
Bass & Turner (1973)	368	.61	2	1	1
Borman et al. (1976)	14	.37	2	1	1
Bruel & Bachner (1961)	65	.73	1	1	1
Bynum et al. (2013)	5278	.29	2	2	1
Campbell (1986) Study 1	141	.48	2	1	1
Campbell (1986) Study 2	154	.51	2	1	1
Campbell (1986) Study 3	113	.53	2	1	1
Campbell (1986) Study 4	149	.49	2	1	1
Campbell (1986) Study 5	154	.58	2	1	1
Campbell (1986) Study 6	126	.63	2	1	1
Campbell (1986) Study 7	143	.59	2	1	1
Campbell (1986) Study 8	156	.62	2	1	1
Cellar et al. (1996)	424	.70	2	1	1
Cummins (1971)	133	.44	1	1	1
Day & Silverman (1989)	43	.49	2	1	1
Dicken & Black (1965)	57	.49	2	1	1
Dunnette & Motowidlo (1976) Study 1	702	.35	1	1	.76
Dunnette & Motowidlo (1976) Study 2	411	.51	1	1	.80
Dunnette & Motowidlo (1976) Study 3	363	.44	1	1	1
Dunnette & Motowidlo (1976) Study 4	204	.48	1	1	1
Farmer (1933) Sample 1	524	.46	1	1	1
Farmer (1933) Sample 2	259	.71	1	1	1
Farmer (1933) Sample 3	347	.50	1	1	1
Farmer (1933) Sample 4	333	.57	1	1	1
Farmer (1933) Sample 5	93	.73	1	1	1
Fineman & Payne (1974)	6	.87	2	1	1
Friedland & Michael (1987)	147	.37	2	1	1
Guion (1965)	48	.75	1	1	1
Gunderson & Nelson (1966)	139	.43	2	1	1
Gunderson & Ryman (1971)	197	.47	2	1	.72
Hilton et al. (1955)	72	.50	1	1	1
Hough (1984)	153	.49	2	1	.55
Hughes & Prien (1986)	49	.42	2	1	1
Ivancevich (1977)	102	.61	2	1	.57
Jurgensen (1944)	40	.83	1	1	1
Landy et al. (1976)	342	.51	2	1	1
Lee et al. (1981)	144	.53	2	1	1

Lowmaster & Morey (2012)	59	.64	1	2	.70
Maxim & Kirchner (1960)	303	.25	2	2	1
Mount et al. (1997)	2297	.43	2	2	1
Robertson et al. (1993)	89	.46	2	1	1
Rothstein (1990) Sample 1	51	.15	2	1	.58
Rothstein (1990) Sample 2	76	.43	2	1	.76
Rothstein (1990) Sample 3	73	.39	2	1	.69
Rothstein (1990) Sample 4	68	.43	2	1	.61
Rothstein (1990) Sample 5	102	.32	2	1	.62
Rothstein (1990) Sample 6	105	.51	2	1	.78
Rothstein (1990) Sample 7	104	.42	2	1	.75
Rothstein (1990) Sample 8	130	.41	2	1	.66
Rothstein (1990) Sample 9	106	.54	2	1	.61
Rothstein (1990) Sample 10	97	.36	2	1	.62
Rothstein (1990) Sample 11	111	.43	2	1	.72
Rothstein (1990) Sample 12	134	.50	2	1	.76
Rothstein (1990) Sample 13	315	.48	2	1	.73
Rothstein (1990) Sample 14	322	.51	2	1	.77
Rothstein (1990) Sample 15	361	.46	2	1	.74
Rothstein (1990) Sample 16	285	.58	2	1	.75
Rothstein (1990) Sample 17	262	.49	2	1	.77
Rothstein (1990) Sample 18	297	.47	2	1	.75
Rothstein (1990) Sample 19	255	.42	2	1	.74
Rothstein (1990) Sample 20	357	.54	2	1	.81
Rothstein (1990) Sample 21	832	.55	2	1	.85
Rothstein (1990) Sample 22	684	.53	2	1	.84
Rothstein (1990) Sample 23	552	.48	2	1	.79
Rothstein (1990) Sample 24	570	.53	2	1	.82
Rothstein (1990) Sample 25	478	.59	2	1	.87
Rothstein (1990) Sample 26	504	.51	2	1	.85
Rothstein (1990) Sample 27	437	.58	2	1	.82
Rothstein (1990) Sample 28	396	.56	2	1	.85
Rothstein (1990) Sample 29	341	.52	2	1	.87
Rothstein (1990) Sample 30	320	.50	2	1	.83
Rothstein (1990) Sample 31	310	.51	2	1	.83
Rothstein (1990) Sample 32	281	.56	2	1	.83
Rothstein (1990) Sample 33	213	.52	2	1	.84
Rothstein (1990) Sample 34	134	.50	2	1	.82
Rothstein (1990) Sample 35	105	.55	2	1	.75
Rothstein (1990) Sample 36	116	.38	2	1	.81
Rothstein (1990) Sample 37	91	.53	2	1	.96
Rush (1953)	100	.51	1	1	.50
Salgado & Blanco (1988)	30	.63	1	1	.65

Salgado et al. (2007)	20	.80	2	1	1
Scullent et al. (2000)	2142	.40	2	2	1
Sprecher (1959)	107	.84	1	1	1
Springer (1953)	100	.65	2	2	1
Stevens & Campion (1986) Study 2	72	.54	2	1	1
Stevens & Campion (1999) Study 1	70	.62	2	1	1
Taylor (1957)	103	.49	2	1	1
Thompson (1970)	71	.42	2	1	1
Van Scotter & Steel (2000) S1	254	.38	2	1	1
Van Scotter & Steel (2000) S2	303	.56	2	1	1
Van Scotter (1994)	192	.50	2	1	.62
Wiley (1976)	244	.49	2	1	1
Worbois (1975)	46	.62	2	1	1

Note. N= sample size; r_{yy}= observed interrater reliability coefficient; Type= mono-item (1), multi-item (2); Purpose= research (1), administrative (2); u= range restriction value.

Appendix 3. Interrater Reliability Coefficients of Supervisory Ratings of Contextual (Citizenship) Performance.

Study	N	r_{yy}	Type	Purpose	*u*
Albrecht, Glaser, & Marks (1964)	31	.62	2	1	1
Bass & Turner (1973)	368	.61	2	1	1
Borman et al. (1976)	14	.50	2	1	1
Bushe & Gibbs (1990)	64	.71	2	1	.77
Bynum et al. (2013)	5278	.30	2	2	1
Carraher et al. (2005) Sample 1	403	.81	1	1	1
Carraher et al. (2005) Sample 2	295	.83	1	1	1
Cellar et al. (1996)	424	.68	2	1	1
Cummins (1971)	133	.44	1	1	1
Day & Silverman (1989)	43	.40	2	1	1
Dicken & Black (1965)	57	.33	2	1	1
Dunnette & Motowidlo (1976) Sample 2	542	.38	1	1	1
Dunnette & Motowidlo (1976) Sample 3	297	.40	1	1	1
Dunnette & Motowidlo (1976) Sample 4	352	.43	1	1	1
Dunnette & Motowidlo (1976) Sample 5	157	.52	1	1	1
Fineman & Payne (1974)	6	.48	2	1	1
Friedland & Michael (1987)	147	.36	2	1	1
Guion (1965)	48	.79	1	1	1
Gunderson & Nelson (1966)	139	.61	2	1	1
Gunderson & Ryman (1971)	228	.64	2	1	1
Hilton et al. (1955)	72	.44	1	1	1
Hogan, Hogan & Busch (1984) Study 1	37	.69	1	1	1
Hogan, Hogan & Busch (1984) Study 2	101	.51	1	1	1
Hough (1984)	153	.45	2	1	.55
Ivancevich (1977)	102	.63	2	1	.57
Landy et al. (1976)	342	.47	2	1	1
Lee, Malone, & Greco (1981)	144	.42	2	1	1
Maxim & Dielman (1987)	303	.23	2	2	1
Mitchell & Albright (1972)	66	.64	2	1	1
Motowidlo et al. (1998)	123	.50	1	1	1
Mount et al. (1997)	2297	.43	2	2	1
Nealy & Owen (1970)	25	.49	2	1	1
Otten & Kahn (1975)	43	.63	2	1	1
Robertson et al. (1993)	89	.64	2	1	1
Rush (1953)	100	.56	1	1	.50
Salgado, Gorriti, & Moscoso (2007)	20	.72	2	1	1
Scullen et al. (2000)	2142	.41	2	2	1
Sprecher (1959)	107	.84	1	1	1

Springer (1953)	100	.66	2	2	1
Thompson (1970)	71	.39	2	1	1
Van Scotter & Motowidlo (1994)	123	.50	1	1	1
Van Scotter (1994)	168	.55	2	1	.59
Woodmansee (1978)	44	.68	2	1	.64
Worbois (1975)	46	.57	2	1	1

Note. N= sample size; r_{yy}= observed interrater reliability coefficient; Type= mono-item (1), multi-item (2); Purpose= research (1), administrative (2); *u*= range restriction value.

Appendix 4. Interrater Reliability Coefficients of Supervisory Ratings of Positive Performance.

Study	N	r_{yy}	Type	Purpose	*u*
Cellar et al. (1996)	424	.68	1	1	1
Day & Silverman (1989)	43	.45	1	1	1
Dunnette & Motowidlo (1976) Sample 1	593	.26	1	1	1
Dunnette & Motowidlo (1976) Sample 2	214	.43	1	1	1
Gunderson & Nelson (1966)	139	.60	2	1	1
Gunderson & Ryman (1971)	228	.57	2	1	1
Lee et al. (1981)	144	.53	2	1	1
Lowmaster & Morey (2012)	59	.43	1	2	.70
Springer (1953)	100	.59	2	2	1
Thompson (1970)	71	.59	1	1	1

Note. N= sample size; r_{yy}= observed interrater reliability coefficient; Type= mono-item (1), multi-item (2); Purpose= research (1), administrative (2); *u*= range restriction value.
