# Supplementary material for: Meta-Analysis of Interrater Reliability of Supervisory Performance Ratings: Effects of Appraisal Purpose, Scale Type, and Range Restriction
Source: Front Psychol. 2019 Oct 18;10:2281. doi: 10.3389/fpsyg.2019.02281 (PMC6813221; doi:10.3389/fpsyg.2019.02281)

Fig. 1. Cumulative Meta-analysis of Research-Purpose Ratings of Overall Job Performance

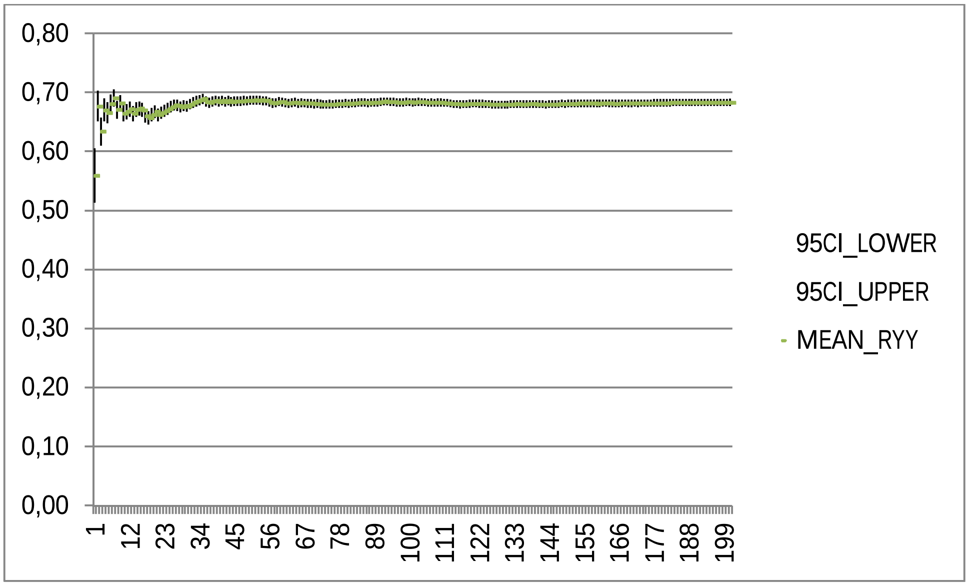

Fig. 2. Cumulative Meta-analysis of Administrative-Purpose Ratings of Overall Job Performance

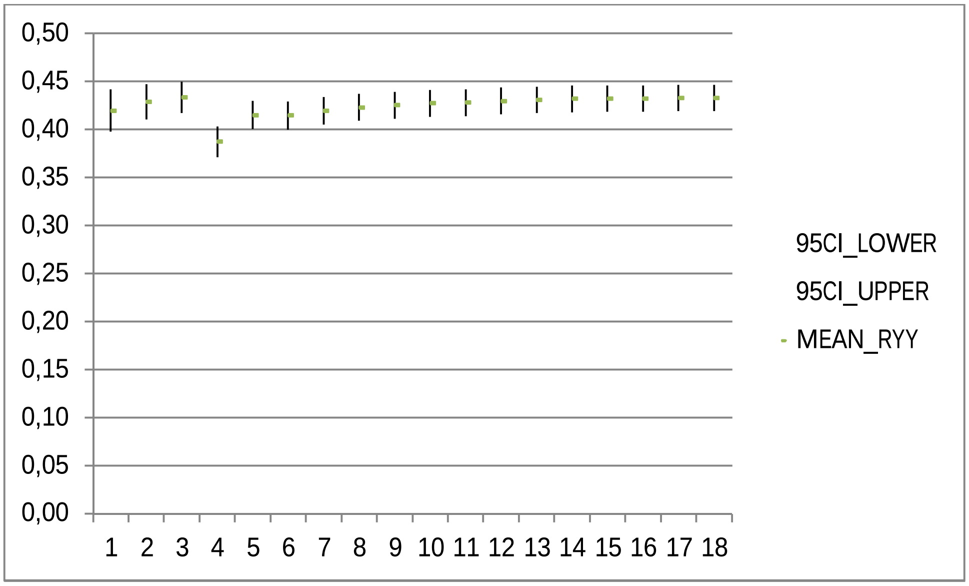

Fig. 3. Cumulative Meta-analysis of Research-Purpose Ratings of Task Performance

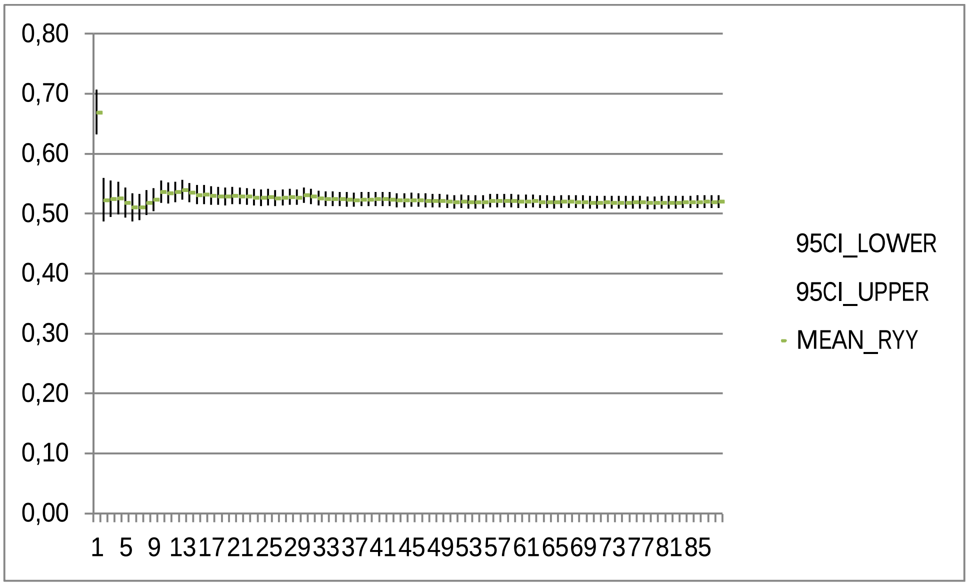

Fig. 4. Cumulative Meta-analysis of Administrative-Purpose Ratings of Task Performance

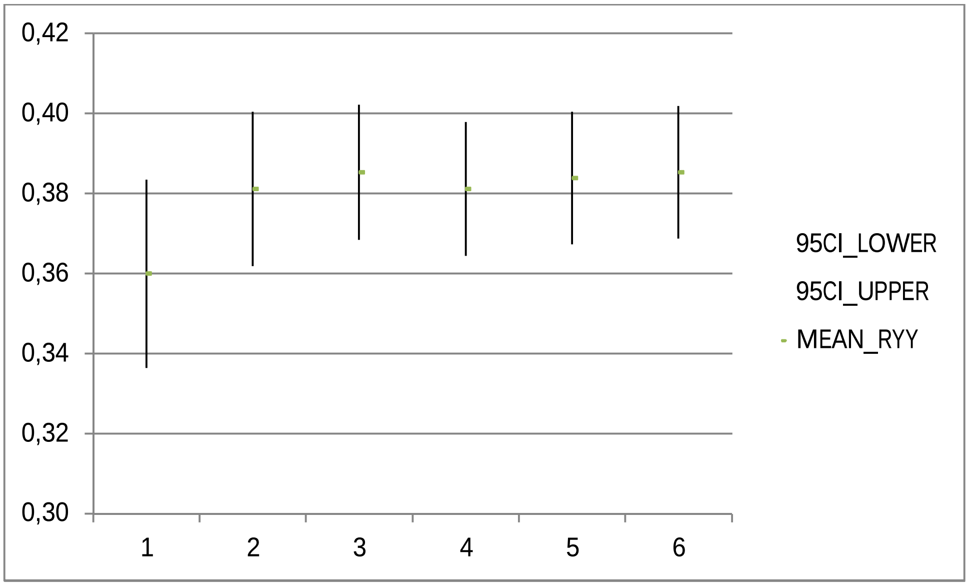

Fig. 5. Cumulative Meta-analysis of Research-Purpose Ratings of Contextual Performance

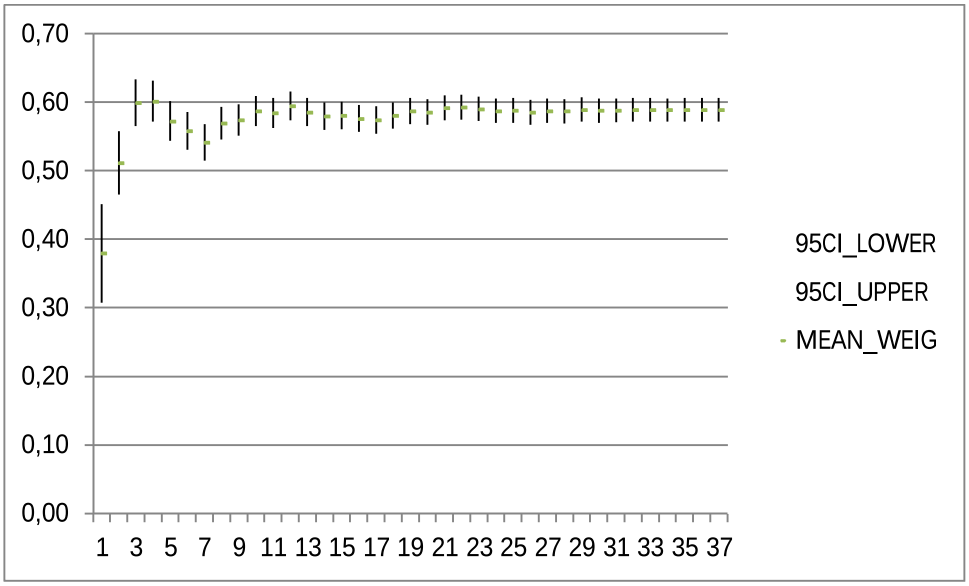

Fig. 6. Cumulative Meta-analysis of Administrative-Purpose Ratings of Contextual Performance

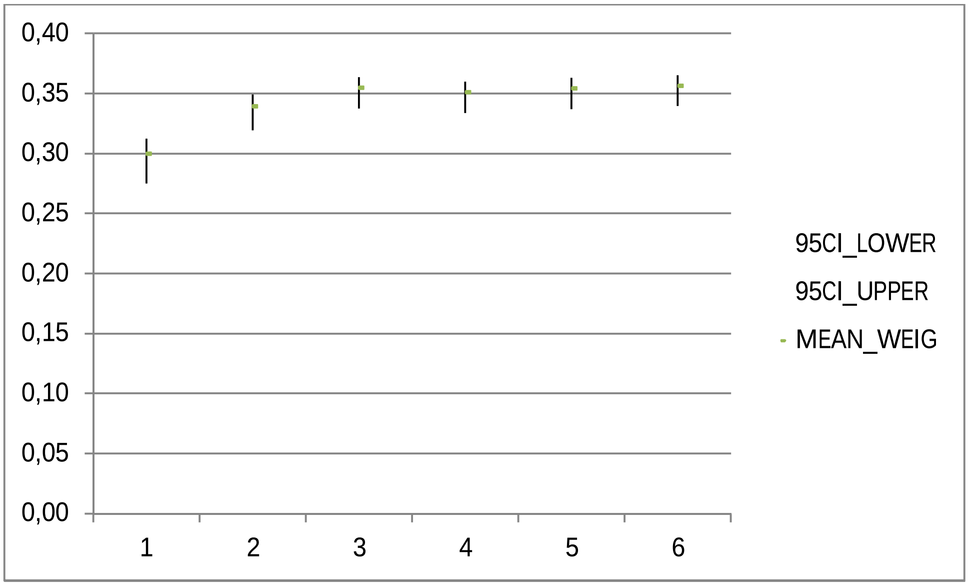

Fig. 7. Cumulative Meta-analysis of Research-Purpose Ratings of Positive Performance

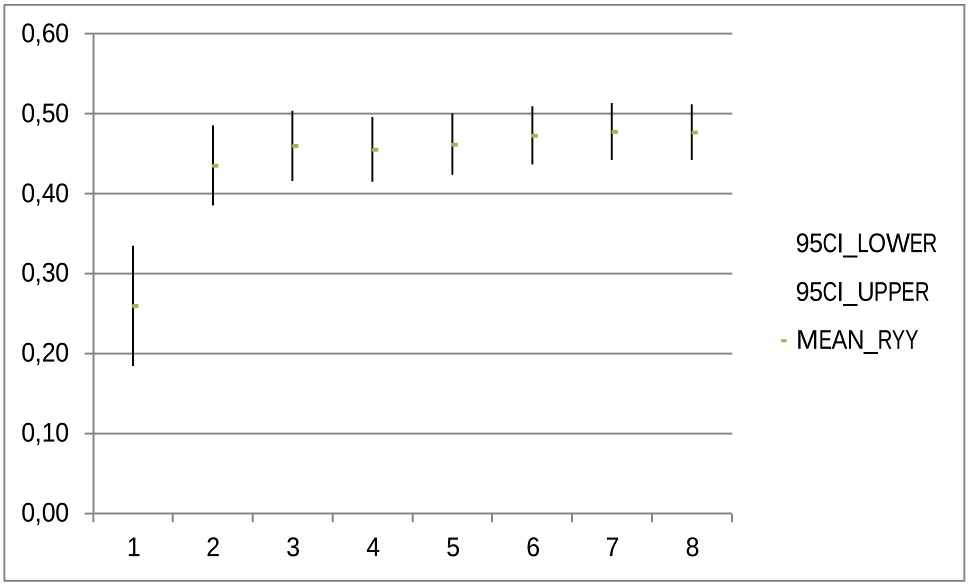

Supplement: Supplementary file 2 [file Data_Sheet_2.PDF]
